# Side-chain-selective deuterium labeling by a simple bio-expression method enhances resolution and simplifies spectra in ^1^H-detected protein solid-state NMR

**DOI:** 10.1007/s10858-025-00476-9

**Published:** 2025-11-01

**Authors:** Yoshiki Shigemitsu, Yuki Miyazaki, Hibiki Terami, Daria Ostapova, Tatsuya Matsunaga, Ryo Takahashi, Takumi Inoue, Toshio Yamazaki, Yoshitaka Ishii

**Affiliations:** 1 School of Life Science and Technology, Institute of Science Tokyo, 4259 Midori-ku, Yokohama, 226-8503 Kanagawa Japan; 2 BDR Center, RIKEN, 1-7-22 Suehiro-cho, Tsurumi-ku, Yokohama, 230-0045 Kanagawa Japan; 3 School of Life Science and Technology, Tokyo Institute of Technology, 4259 Midori-ku, Yokohama, 226-8503 Kanagawa Japan

**Keywords:** Isotope labeling, Selective protonation, Medium-switching, Solid-state NMR

## Abstract

We present a novel approach for side-chain-selective deuteration of proteins to improve ^1^H_α_ spectral resolution and to simplify side-chain signals in ^1^H-detected protein solid-state NMR (SSNMR) with a simple bio-expression method using *E. coli* BL21 (DE3). ^1^H-detected SSNMR using ultra-fast magic-angle spinning (MAS) at a spinning rate of 60 kHz or higher is attracting attention as a powerful method of protein structure determination. However, even with ultra-fast MAS at 100 kHz, the ^1^H line broadening due to ^1^H-^1^H dipolar interactions cannot be eliminated, posing an obstacle to signal assignment and structure determination. To improve resolution for SSNMR-based protein structural analysis, we developed a method to selectively deuterate side-chains at a high deuteration level while maintaining the protons at the α-position. This selective labeling method is based on the transamination reaction in the amino-acid biosynthesis pathway and switching a medium from an unlabeled H_2_O medium containing D-glucose (glucose), ammonium chloride, and amino acid mixture for rapid cell growth to a labeled H_2_O medium containing [^2^H, ^13^C]-glucose, ^15^N-labeled ammonium chloride, and a [^2^H, ^13^C, ^15^N]-labeled amino-acid mixture just before the induction. With [^2^H, ^13^C]-labeled glucose and a [^2^H, ^13^C, ^15^N]-labeled amino-acid mixture as the carbon sources, this medium-switching method provides a simple and efficient means to express a selectively deuterated protein GB1 domain (GB1) sample, which is achieved by promoting efficient back-protonation at the α-position via the transamination reaction while retaining side-chain deuterons to a large extent. The yield of the GB1 protein was found to be enhanced by a factor of ca. 1.5 with the medium-switching method, compared with that for the expression with a traditional M9 minimal medium in H_2_O without medium-switching. For the selectively deuterated GB1 sample, the resultant ^1^H resolution for resolved ^1^H_α_ peaks in ^1^H-detected 2D ^1^H/^13^C correlation SSNMR at a MAS rate of 70 kHz was improved by a factor of 1.21 on average, compared with the corresponding resolution for a fully protonated, uniformly ^13^C- and ^15^N-labeled GB1 sample. Furthermore, side-chain signal assignment is facilitated by utilizing residual protons of the side chains. Our results also suggest that the side-chain deuteration level can be altered by adjusting the level of the deuterated amino-acid mixture in the expression system.

**Supplementary Information:**

The online version contains supplementary material available at 10.1007/s10858-025-00476-9.

## Introduction

Solid-state NMR (SSNMR) using ^1^H detection (Ishii and Tycko 2000; Ishii et al. 2001) is attracting attention as a powerful method of signal assignment and protein structure determination for mass-limited non-crystalline and insoluble protein samples. However, limited ^1^H resolution due to strong ^1^H-^1^H dipolar interactions between the dense proton network is a major challenge for structural determination, particularly for large proteins. Ultra-fast magic-angle spinning (MAS) beyond a spinning rate of 60 kHz is a powerful strategy to increase resolution, as it removes dipolar couplings and other anisotropic spin interactions (Andreas et al. 2015). However, the ^1^H line broadening due to ^1^H-^1^H dipolar interactions cannot be fully eliminated, even with ultra-fast MAS at a spinning rate of ~ 100 kHz (Penzel et al. 2019; Wickramasinghe et al. 2015).

Another approach for enhancing ^1^H spectral resolution is to replace ^1^H in a protein with ^2^H to minimize ^1^H-^1^H dipolar interactions (LeMaster and Richards 1988; Zhang and Ingen 2016). Amide proton back-exchange in deuterated proteins (Löhr et al. 2003; Nieuwkoop et al. 2015; Feng et al. 2006) and observing residual ^1^H resonances in highly per-deuterated proteins (Asami et al. 2010; Chevelkov et al. 2006) are both simple and effective ^2^H-labeling methods for ^1^H-detected SSNMR. However, the former method does not provide sufficient distance restraint information for the side-chains, while the latter suffers from poor sensitivity due to limited proton density. More advanced deuteration schemes that achieve stereospecific deuteration potentially solve these problems. Stereo-specific deuteration of amino-acids leads to the narrowing of ^1^H line widths of proteins due to reduced ^1^H-^1^H dipolar interactions. Stereo-array isotope labeling (SAIL) amino acids are more widely used not only with cell-free expression systems but also with *E. coli-*based expression systems to produce stereo-specific deuterated proteins (Kainosho et al. 2006; Schmidt et al. 2014; Miyanoiri et al. 2013). Although this technique was initially only used for solution NMR, its application to SSNMR analysis of amino acids and proteins has also been reported to narrow ^1^H line widths and improve sensitivity (Wang et al. 2015; Takahashi et al. 2010). Nevertheless, the need for stereo-specific ^2^H-labeled amino acids often makes SAIL labeling costly.

One effective approach for selective deuteration of a protein sample is to use transamination and other enzymatic reactions for amino-acid synthesis in *E. coli* in a constructive manner. Transamination by transaminase is essential in the biosynthesis of any amino acids. In the transamination, an amino acid is converted to an α-keto acid. Then, the α-keto acid accepts an amino group derived from another amino acid to be converted back to the original amino acid. In this process, solvent-derived protons or deuterons can be introduced into the α-position of the amino acid. For expression of fully deuterated proteins, unless high levels of D_2_O in the medium are utilized, deuterons attached to carbon in amino acids are replaced by protons from H_2_O, which are incorporated into a pathway for amino-acid synthesis, resulting in unfavorable scrambling (Tonelli et al. 2011). The scrambling of H_α_ in Gly and H_β_ in Ala, Asp, and Asn is introduced by transaminase, while that of H_γ_ in Gln and Glu is introduced by glutamine synthetase. Stereospecific deuteration was recently achieved for Asp, Asn, Lys, and Met amino-acid residues using an *E. coli* bio-expression system with rhamnose, pyruvate, and fumarate by switching H_2_O medium to D_2_O just before induction (Danmaliki et al. 2017). Rhamnose was used as a carbon source instead of glucose to achieve higher side-chain deuteration at aromatic amino acids than glucose. Pyruvate and fumarate were supplied as products of the glycolytic system and tricarboxylic acid (TCA) cycle. These enzymatic reactions are stopped by inhibitors, as described below. This method takes advantage of replacing of ^1^H_α_ in the amino acids with ^2^H_α_ by aspartate transaminase in combination with the addition of oxalate and malonate, which inhibit key metabolic pathways that produce scrambling in the TCA cycle. This approach does not require SAIL-labeled amino acids, but it is only effective for introducing ^2^H to the side chains of specific amino acids such as Asp, Asn, Lys, and Met. Recently, Movellan et al. reported an excellent method to introduce protons selectively at the α-position in deuterated amino acids based on the transamination reaction using an α-keto acid mixture derived from a deuterated amino-acid mixture pretreated with L-amino-acid oxidase and bovine liver catalase (Movellan et al. 2019). Using the treated amino-acid mixture in the medium, ^1^H_α_-protonated protein with some scrambled side-chain ^1^H was prepared with an *E. coli* expression system. Although this succeeded in improving the ^1^H_α_ resolution of protein SSNMR, this method requires extra inconvenient steps for treating the amino-acid mixture with the enzymes, which may prevent widespread usage of this method.

**Fig. 1:**
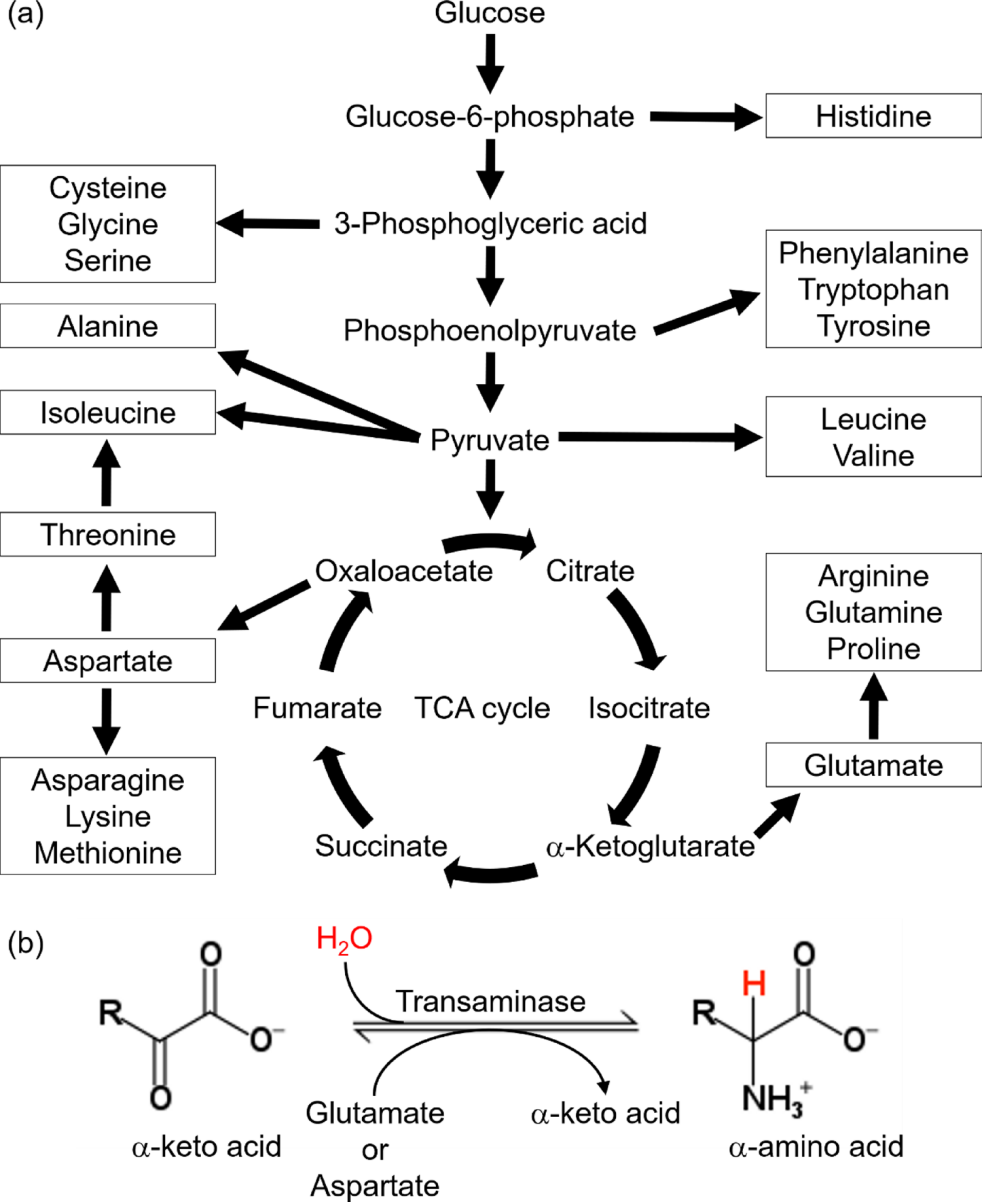
(**a**) Schematic of amino-acid biosynthesis pathways in the *E. coli* expression system when glucose is used as a carbon source. (**b**) Transamination reaction is essential for amino-acid biosynthesis. In this reaction, hydrogen derived from the solvent is introduced into the α-position of α-keto acids, which is an amino-acid precursor

**Fig. 2:**
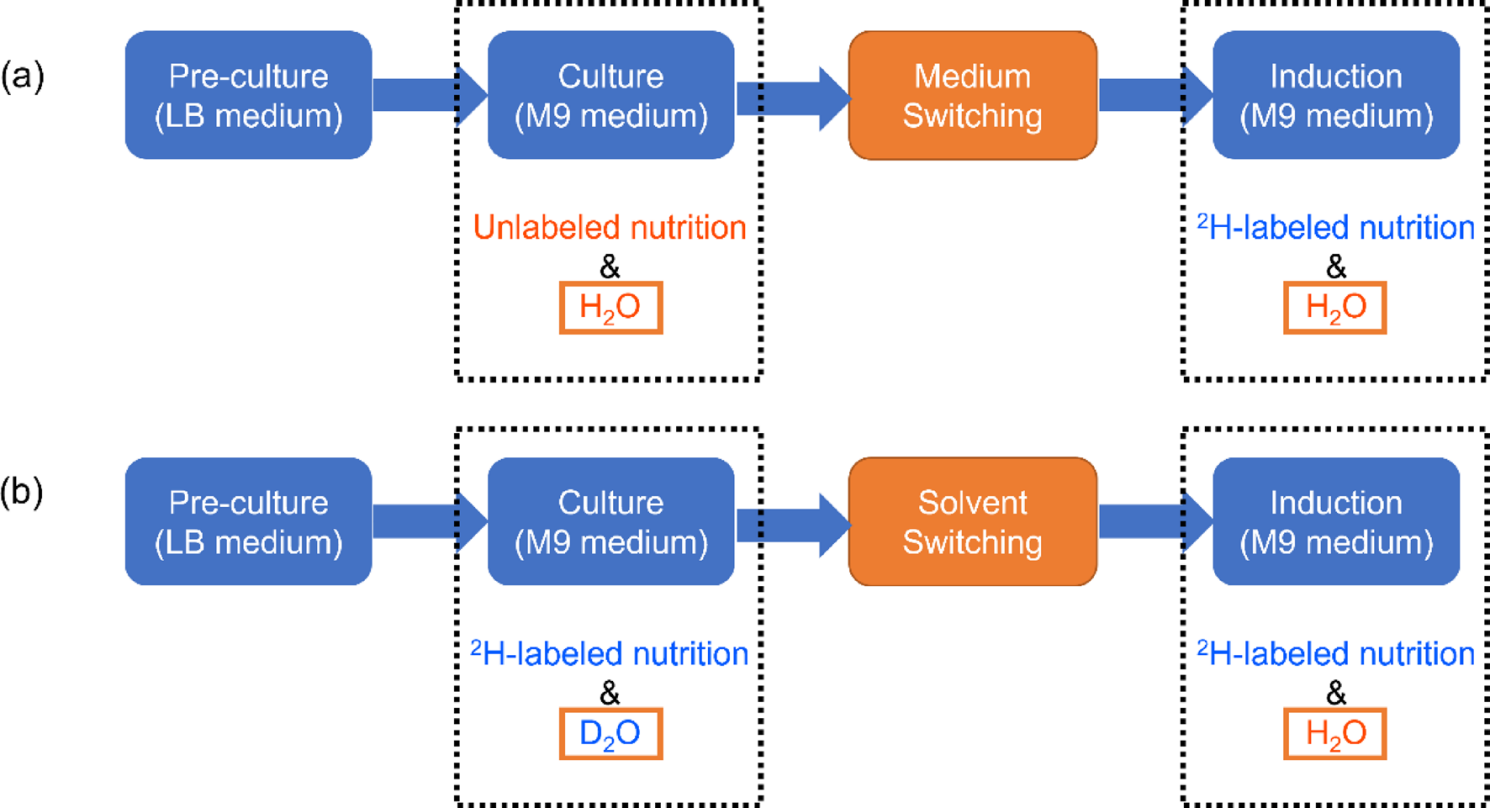
Schematic of the (**a**) medium-switching and (b) solvent-switching method. For both methods, after medium/solvent switching, ^2^H are incorporated from ^2^H nutrients into the deuterated amino-acid precursors and ^2^H in the deuterated amino-acid precursors is replaced by ^1^H from H_2_O in the medium by transaminase, resulting in protonation at the α-position as shown in Fig. 1b. For the medium switching, an unlabeled medium is switched to a ^2^H-labeled medium dissolved in H_2_O (i.e., with ^2^H-labeled glucose and amino acids). For the solvent-switching method, a ^2^H-labeled medium dissolved in D_2_O (with glucose and amino acids) is switched to a ^2^H-labeled medium dissolved in H_2_O

In this work, to improve resolution in ^1^H-detected SSNMR by deuteration of side chains for the analysis of protein backbone structure, we developed a simple method based on an *E. coli* expression system to introduce side-chain selective deuteration at a high deuteration level, while maintaining the protons at the α-position by taking advantage of the transamination reaction. Based on our knowledge of metabolism and amino-acid biosynthesis pathways (Fig. 1a) and the transamination reaction (Fig. 1b), we formulated a hypothesis that high levels of side-chain-selective deuteration of protein samples for SSNMR analysis can be achieved by introducing protons into the α-position of deuterated α-keto acids from a deuterated amino-acid mixture using the transamination reaction. To label the metabolic precursors with deuterium at a high level, we propose switching medium from an unlabeled H_2_O medium containing glucose, ammonium chloride, and amino-acid mixture for rapid cell growth to a labeled H_2_O medium containing [^2^H, ^13^C]-glucose as a carbon source, ^15^N-labeled ammonium chloride as a nitrogen source, and, a [^2^H, ^13^C, ^15^N]-labeled amino-acid mixture to accelerate cell growth and protein production just before the induction (Fig. 2a). We demonstrate that we can successfully protonate all the backbone ^13^C_α_ at a high level while high deuteration rate of the side-chains (~ 66%) with the medium-switching method. Most notably, nearly complete deuteration was observed for non-exchangeable aliphatic hydrogens in Ile, Leu, Phe, Trp, Tyr, and Val. Since our original idea was to switch solvent from D_2_O to H_2_O in the process, we also tested an alternative approach of culturing cells with D_2_O minimal labeled medium containing ^15^N-labeled ammonium chloride, [^2^H, ^13^C]-labeled glucose, and an [^2^H, ^13^C, ^15^N]-labeled amino-acid mixture and switching the medium to an H_2_O minimal medium containing the same labeled materials immediately before induction, as described below (Fig. 2b). Amide hydrogen can also be back-exchanged from ^2^H to ^1^H by the solvent-switching method. We also report that side-chain deuteration efficiency by the solvent-switching method (67%) is comparable to that of the medium-switching method described above. This medium switching method is likely to offer cost-effective means to enhance resolution of ^1^H-detecetd protein SSNMR spectra by side-chain selective deuteration.

## Materials and methods

For expression, sodium phosphate dibasic (Na_2_HPO_4_, catalog number: 28-3750 for yield comparison, and 795410 for the other experiments), potassium dihydrogen phosphate (KH_2_PO_4_, 24-5260), sodium chloride (NaCl, 28-2270), magnesium sulfate (MgSO_4_, 746452), calcium chloride (CaCl_2_, 746495), ampicillin sodium salt (A9518), biotin (B4501), thiamine hydrochloride (T4625), and isopropyl β-D-1-thiogalactopyranoside (IPTG, 15-2461) were purchased from Sigma-Aldrich. As nitrogen and carbon sources, ammonium chloride (A4514) and glucose (07-0680) for unlabeled protein were also purchased from Sigma-Aldrich. For isotope-labeled protein, ^15^N-ammonium chloride, ^13^C-labeled glucose, and [^2^H, ^13^C]-labeled glucose were purchased from Cambridge Isotope Laboratories (NLM-467), Chlorella Industry, and ISOTEC (1552151), respectively. D_2_O was purchased from Cambridge Isotope Laboratories (DLM-4-1000) and ISOTEC (151882). For the amino-acid mixture, we purchased unlabeled, and [^2^H, ^13^C, ^15^N]-labeled Celtone Base Powder (Celtone) from Cambridge Isotope Laboratories (CGM-1030P-U for unlabeled, and CGM-1030P-CDN for [^2^H, ^13^C, ^15^N]-labeled). Cobalt (II) chloride hexahydrate (CoCl_2_∙6H_2_O, 09206-92), copper (II) chloride dihydrate (CuCl_2_∙2H_2_O, 09505-01), iron (II) sulfate heptahydrate (FeSO_4_, 19531-25), boric acid (H_3_BO_3_, 05218-62), manganese (II) chloride tetrahydrate (MnCl_2_∙4H_2_O, 21211-32), hexaammonium heptamolybdate tetrahydrate ((NH_4_)_6_MO_7_O_24_·4H_2_O, 02521-62), and zinc sulfate heptahydrate (ZnSO_4_·7H_2_O, 37011-91) were purchased from Nacalai Tesque. Ethylenediamine-*N*,* N*,*N’N’*-tetraacetic acid, disodium salt, dihydrate (disodium EDTA∙2H_2_O, 343-01861) was purchased from DOJINDO. For protein purification, tris(hydroxymethyl)aminomethane (Tris) was purchased from Sigma-Aldrich (30-5000) and Nacalai Tesque (35434-76), NaCl was purchased from FUJIFILM Wako (191-01665), and hydrochloric acid (18321-05) for pH adjustment was purchased from Nacalai Tesque. We used an NGC Quest 10 chromatography system (Bio-Rad) and HiTrap Q HP column (Cytiva) for protein purification. For buffer exchange, in addition to Na_2_HPO_4_ described above, NaH_2_PO_4_ (28-3809) was purchased from Sigma-Aldrich. Amicon Ultra-15 centrifuge filter units (MWCO: 3 kDa) were purchased from Merck-Millipore (UFC900308, UFC900324, or UFC900396). For SSNMR, 2-propanol (IPA, 34863) and 2-methylpentane-2,4-diol (MPD, 68340) to make GB1 microcrystals, and ethylenediaminetetraacetic acid copper (II) disodium salt (Cu-EDTA, 03668) as a paramagnetic dopant, were purchased from Sigma-Aldrich.

### Production of fully protonated Immunoglobulin G-binding protein G, β1 domain (GB1) in the ^1^H-nutritions media

Fully protonated, uniformly ^13^C- and ^15^N-labeled ([^13^C, ^15^N]-) T2Q and F52Y mutants of GB1 protein were produced as previously described (Li et al. 2013). Briefly, *E. coli* cells were grown overnight in an LB-agar plate containing 50 µg/mL ampicillin at 37 ℃. A single colony was transferred to 5 mL of LB medium containing 50 µg/mL ampicillin in a 15-mL conical tube and grown at 120 rpm and 37 ℃ using a MAXQ 4000 shaker incubator (Thermo Scientific) until OD_600_ reached 0.5−0.8. Then, 20 µL of the medium containing the cells was inoculated to 200 mL of M9 media in a 500-mL Erlenmeyer flask; the M9 media contained 6.8 g/L of Na_2_HPO_4_, 3.0 g/L of KH_2_PO_4_, 0.5 g/L of NaCl, 0.5 g/L of ^15^N-ammonium chloride, 2.0 g/L of ^13^C-glucose, 2 mM MgSO_4_, 0.1 mM CaCl_2_, and 50 µg/mL ampicillin, all of which were dissolved in H_2_O. The cells were grown at 120 rpm and 37 ℃ until OD_600_ ≈ 0.8. Then, GB1 was induced with 1 mM IPTG, and the cells were grown for 3 h after the induction. The cells were harvested by centrifuging the culture at 4,000 ×g for 20 min at 4 ℃ with an Avanti J-26 S XP centrifuge machine and a JLA-16.250 fixed angle rotor (Beckman-Coulter).

### Production of GB1 in ^2^H-nutrition media with medium-switching with a deuterated amino-acid mixture

We describe a medium-switching method (i.e. from unlabeled H_2_O medium to [^2^H, ^13^C, ^15^N]-labeled H_2_O medium), which was employed with a deuterated amino-acid mixture. To produce side-chain-selectively deuterated and [^13^C, ^15^N]-labeled GB1 protein, cells were initially grown overnight in an LB-agar plate (made with H_2_O) containing 50 µg/mL ampicillin at 37 ℃. A single colony was transferred to 5 mL of LB medium containing 50 µg/mL ampicillin in a 15-mL conical tube and grown at 120 rpm and 37 ℃ until OD_600_ reached 0.5−0.8. Then, 20 µL of the medium containing the cells was inoculated to 200 mL of M9 media in a 500-mL Erlenmeyer flask; the M9 media contained 6.8 g/L of Na_2_HPO_4_, 3.0 g/L of KH_2_PO_4_, 0.5 g/L of NaCl, 0.5 g/L of ammonium chloride, 2.0 g/L of glucose, 1.0 g/L of unlabeled Celtone, 2 mM MgSO_4_, 0.1 mM CaCl_2_, and 50 µg/mL ampicillin, all of which were dissolved in H_2_O. The cells were grown at 120 rpm and 37 ℃ until OD_600_ ≈ 0.8, and then harvested at 4,000 ×g for 20 min at 4 ℃ using an Avanti J-26 S XP centrifuge machine and a JLA-16.250 fixed angle rotor with adaptors for conical tubes. The harvested cells were resuspended in 200 mL of H_2_O M9 media supplemented with ^2^H-nutritions in a 500-mL Erlenmeyer flask just before the induction; the H_2_O M9 media contained 6.8 g/L of Na_2_HPO_4_, 3.0 g/L of KH_2_PO_4_, 0.5 g/L of NaCl, 0.5 g/L of ^15^N-labeled ammonium chloride, 2.0 g/L of [^2^H, ^13^C]-labeled glucose, 1.0 g/L of [^2^H, ^13^C, ^15^N]-labeled Celtone, 2 mM MgSO_4_, 0.1 mM CaCl_2_, and 50 µg/mL ampicillin, all of which were dissolved in H_2_O. A schematic of the medium-switching method is shown in Fig. 2a. After production of GB1 was induced with 1 mM IPTG, the cells were cultured for 3 h and then harvested by centrifugation at 4,000 ×g for 20 min at 4 ℃.

### Production of GB1 in ^2^H-nutrition media with solvent-switching with/without a deuterated amino-acid mixture

We also prepared [^2^H, ^13^C, ^15^N]-labeled GB1 protein by the solvent-switching method from D_2_O to H_2_O. In this procedure, a mixture of ^15^N-labeled ammonium chloride, [^2^H, ^13^C]-labeled glucose, and [^2^H, ^13^C, ^15^N]-labeled Celtone in D_2_O was added to the medium for the first half of the protocol for cell growth, while a mixture of ^15^N-labeled ammonium chloride, [^2^H, ^13^C]-labeled glucose, and [^2^H, ^13^C, ^15^N]-labeled Celtone in H_2_O was added for the second half just before the induction (Fig. 2b). When the OD_600_ in 5 mL LB medium in *E. coli* cells reached 0.5−0.8, the cells harvested at 4,000 ×g for 20 min at 4 °C using an Avanti J-26 S XP centrifuge machine and a JLA-16.250 fixed angle rotor. The harvested *E. coli* cells were resuspended in 200 mL of filtered stable isotope-labeled nutrient and D_2_O M9 medium in a 500-mL Erlenmeyer flask. Other conditions are the same as in the previous section. For comparison, we also expressed [^2^H, ^13^C, ^15^N]-labeled GB1 protein with the solvent-switching method but without the deuterated amino-acid mixture. The protocol was the same as described in the previous paragraph, except that neither unlabeled Celtone nor [^2^H, ^13^C, ^15^N]-labeled Celtone was added to the D_2_O/H_2_O M9 medium for solvent-switching. For the evaluation of the yields, trace element mixtures in Table S1 were added in place of 0.1 mM CaCl_2_ in M9 medium for better reproducibility (see the caption of Fig. S3 about the details).

### Purification of GB1

The purification of the GB1 samples was performed as previously described (Li et al. 2013) with minor modifications. Briefly, the cell pellet was resuspended in 10−15 mL of 20 mM Tris-HCl buffer (pH 8.0). Then, the sample was heated at 80 ℃ for 10 min to disrupt cell membranes and cell walls. After the lysate was centrifuged at 20,000 ×g for 10 min or 10,000 ×g for 20 min at 4 ℃ using an Avanti J-26 S XP centrifuge machine equipped with fixed angle rotors (Beckman-Coulter), the supernatant was collected and filtered using a 0.22-µm syringe filter. The filtrate was loaded onto a 5-mL HiTrap Q HP column and equilibrated with 20 mM Tris-HCl (pH 8.0). Using a linear NaCl gradient from 0−1.0 M in the same buffer, GB1 was eluted at around 70 mM NaCl. The identity and purity of the protein were verified using MALDI-TOF-MS with an ultrafleXtreme mass spectrometer (Bruker Daltonics). The mass spectrometer was calibrated using Peptide Standard II and Protein Calibration Standard I (Bruker Daltonics).

### Crystallization of GB1 protein

The preparation of microcrystalline GB1 samples was performed as previously described (Franks et al. 2005) with slight modifications. Briefly, the eluted fractions were concentrated using a 3 kDa MWCO filter unit, and the buffer was exchanged to 50 mM sodium phosphate (pH 5.5). The final protein concentration was 30 mg/mL. After the concentrated GB1 solution was transferred to a microtube, an equal volume of a solution consisting of IPA and MPD in a 1:2 (*v/v*) ratio was added three times to precipitate the protein. For example, for 100 µL of a protein solution, we added 100 µL of IPA/MPD solution to it and mixed the solution by repeated inversions. We repeated this operation one more time. Finally, we added another 100 µL of IPA/MPD solution to the protein solution and left it to stand in fridge overnight. After confirming the formation of microcrystals, the solution was centrifuged using a table-top centrifuge unit at 4,000 ×g for 5 min at room temperature. The supernatant was transferred to another microtube, and the volume was checked. Then, Cu(II)-EDTA powder, a paramagnetic dopant to shorten longitudinal relaxation time (Wickramasinghe et al. 2007), was dissolved in the supernatant at a concentration of 10 mM. Then, the microcrystalline sample was incubated with the supernatant containing the paramagnetic dopant and stored at 4 ℃ until packing. The GB1 microcrystalline sample was packed in a 1-mm rotor (JEOL) by ultracentrifugation using a home-built device for SSNMR experiments.

### Solution NMR spectroscopy

All the GB1 samples used for the solution NMR experiments were ~ 0.1 mM in protein concentration and 500 µL in volume. The buffer conditions were 50 mM sodium phosphate in D_2_O at pH 5.5. All the measurements were performed at 298 K on a Bruker Avance III 600 MHz spectrometer equipped with a cryoprobe. We set the recycle delay to 6 s to account for the effect on relaxation time of the side-chain deuteration. The ^1^H *T*_1_ values for the GB1 samples were identified to be in a range of 0.8−1.2 s. All experimental data were processed in NMRPipe/NMRDraw software (Delaglio et al. 1995). ^1^H and ^13^C chemical shift assignments for the aliphatic region in the 2D ^1^H-^13^C HSQC spectra of GB1 were obtained based on published assignments (Ikeya et al. 2016), and the relevant peak intensities were measured from the data with the error ranges estimated from the noise intensities.

### SSNMR spectroscopy

The fully protonated and partially deuterated [^13^C, ^15^N]-labeled GB1 microcrystalline samples were packed into 1-mm rotors (JEOL). The total mass of the sample packed in each rotor was in a range of 0.4−1.1 mg, of which ~ 60% was protein. All the measurements were performed at 298 K on a 900 MHz JEOL spectrometer, and ^1^H detection was adopted using 70 kHz MAS and a paramagnetic-assisted condensed data collection method (Parthasarathy et al. 2013; Wickramasinghe et al. 2009) with a SLAP scheme for solvent suppression (Matsunaga et al. 2021). All the experimental data were processed and analyzed using NMRPipe/NMRDraw software. ^13^C and ^1^H chemical shifts were referenced to DSS using adamantane as a secondary standard (Harris et al. 2008). ^1^H line widths were obtained by analyzing ^1^H-detected 2D ^13^C/^1^H chemical-shift correlation spectra. Signal assignments were based on published assignments (Andreas et al. 2016).

## Results and discussion

### Characterization of GB1 protein obtained by the medium-switching method

**Fig. 3:**
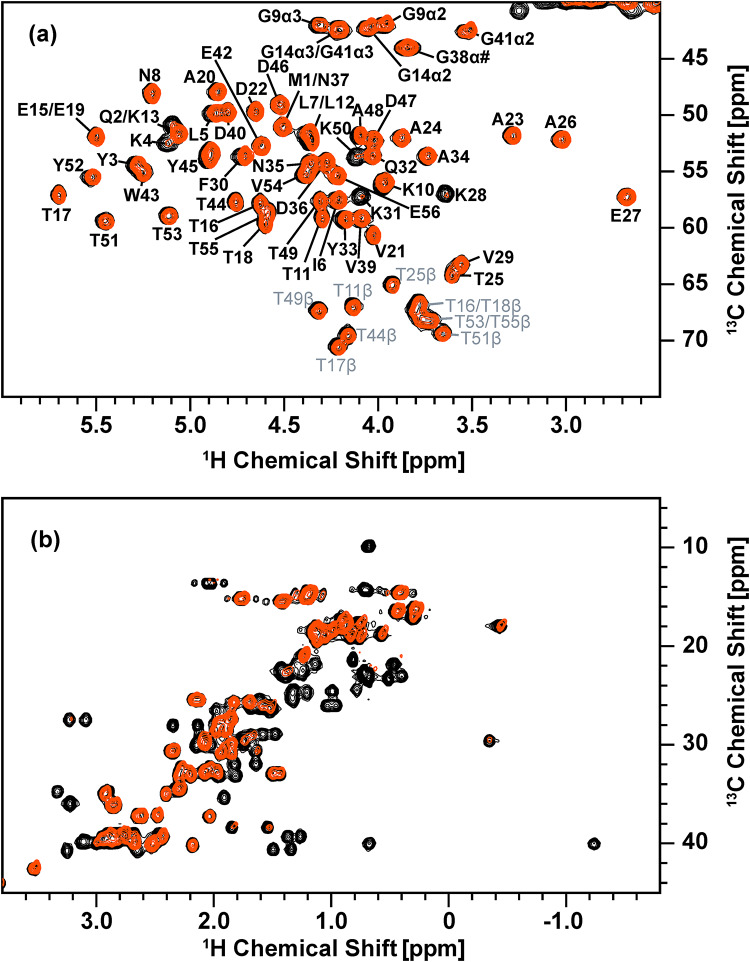
Overlay of ^1^H-^13^C HSQC solution NMR spectra of fully protonated GB1 (black) and selectively deuterated GB1 produced by the medium-switching method with the addition of the unlabeled amino-acid mixture in the medium before switching the medium and with that of the deuterated amino-acid mixture after switching the medium (red) in (a) ^1^H_α_/^13^C_α_ and (b) the side-chain aliphatic region. Both samples were dissolved in 50 mM phosphate buffer (pH 5.5), and these spectra were obtained with a 600 MHz solution NMR spectrometer equipped with a cryoprobe. The fully protonated and selectively deuterated GB1 protein concentrations were 83 and 96 µM, respectively. Contour levels were adjusted based on the protein concentration for each sample to compare signal intensities

We first quantified the selective protonation achieved with the proposed selective-deuteration method involving medium-switching from an unlabeled H_2_O medium to a labeled H_2_O medium with ^15^N-labeled ammonium chloride, [^2^H, ^13^C]-labeled glucose, and [^2^H, ^13^C, ^15^N]-labeled amino-acid mixture using solution NMR for GB1. Figure 3 shows a comparison of the 2D ^1^H-^13^C HSQC spectra of selectively deuterated [^13^C, ^15^N]-labeled GB1 produced with the [^2^H, ^13^C, ^15^N]-labeled amino-acid mixture (red) and fully protonated [^13^C, ^15^N]-labeled GB1 (black) in (a) the ^1^H_α_/^13^C_α_ region and (b) the aliphatic side-chain region. The control spectrum in black serves as a reference (see Fig. S1 for assignments of the side-chain aliphatic region), to which deuteration-labeling efficiency was quantified for the GB1 sample produced by the medium-switching deuterium-labeling method. For the deuterated [^13^C, ^15^N]-labeled GB1 sample produced with the amino-acid mixture, except for attenuated signals of Lys, signals for H_α_ atoms were maintained for all the residues in the selectively deuterated sample as we hypothesized (Fig. 3a). Furthermore, in the aliphatic region, about half of the side-chain peaks observed in fully protonated [^13^C, ^15^N]-labeled GB1 were eliminated in the selectively deuterated sample (Fig. 3b).

For more quantitative analysis, the peak intensities for the HSQC spectrum of the selectively deuterated GB1 were compared with those of the fully protonated [^13^C, ^15^N]-labeled GB1 to determine the level of deuteration for the ^1^H-^13^C pairs of each amino-acid. Table 1 summarizes the peak intensities for each amino acid of the selectively deuterated [^13^C, ^15^N]-labeled GB1 normalized by the corresponding peak intensities of fully protonated [^13^C, ^15^N]-labeled GB1. For the 2D ^1^H_α_/^13^C_α_ correlation data in Figs. 3a, and 48 well-resolved peaks in 56 amino-acid residues were analyzed. It should be noted that the peak intensities of the selectively deuterated sample used in the calculation were normalized by a scaling factor, which is a ratio of the concentration of the selectively deuterated sample to that of the fully protonated sample. Quantitative analysis by intensity comparison (excluding overlapped signals) showed that except for Asn, Ile, Lys, Thr, and α2 of Gly, all other amino acids showing resolved signals (see Table 1) indicate high levels of relative ^1^H_α_ signal intensities above ~ 90%, compared with the corresponding signals of the fully protonated GB1 sample. For Asn, Ile, Lys, Thr, and α2 of Gly, the relative ^1^H_α_ signal intensities were 77%, 72%, 27%, 61%, and 47%, respectively. On average, the relative ^1^H_α_ signal intensities for all the residues in the selectively deuterated protein were ~ 90%. It should be noted that for some amino acids, signal narrowing effects due to the side-chain deuteration were likely to introduce relative intensity higher than 100% (see Table 1).

**Table 1:** Relative peak intensities for the aliphatic region in a 2D ^1^H-^13^C HSQC solution NMR spectrum of GB1 prepared by the medium-switching from H_2_O medium containing the unlabeled amino-acid mixture to H_2_O medium containing the deuterated amino-acid mixture

Amino-acid type	Normalized signal intensities attributed to a chemical group (%)				
	α	β	γ	δ	ε
Alanine	103.9 ± 1.5	37.7 ± 0.6			
Asparagine	76.9 ± 2.5	31.0 ± 2.0			
Aspartate	115.4 ± 2.1	78.7 ± 2.6			
Glutamate	107.4 ± 1.9	73.8 ± 1.7	43.3 ± 1.2		
Glutamine	99.8 ± 4.0	77.0 ± 3.9	40.8 ± 1.9		
Glycine	α2: 47.2 ± 2.1 α3: 88.2 ± 2.7				
Isoleucine	71.7 ± 3.3	0	γ1: 0 γ2: 0	0	
Leucine	106.0 ± 2.7	0	0	0	
Lysine	26.5 ± 1.2	0	0	9.1 ± 0.4	18.8 ± 0.8
Methionine^1)^	*	111.2 ± 9.4	0	-	0
Phenylalanine	140.2 ± 6.1	0			
Threonine	60.8 ± 1.0	43.4 ± 0.8	36.8 ± 0.5		
Tryptophan^1)^	*	14.0 ± 0.8			
Tyrosine	126.2 ± 3.2	0			
Valine	97.5 ± 2.1	16.3 ± 0.6	8.2 ± 0.2		

1) For these amino acids, signals indicated by * could not be measured due to signal overlap with other peaks

The intensities listed for each amino-acid type are normalized by the corresponding intensities of fully protonated GB1. To adjust differences in the sample concentrations, the peak intensities of the deuterated sample used in the calculations are normalized by a scaling factor, which is the concentration of the deuterated sample divided by the concentration of the fully protonated sample. In cases where multiple residues having the same amino-acid type are present, the average values are indicated. Errors were calculated by error propagation with noise

It is noteworthy that the method improved the protein yield (58 ± 3 mg/L culture), compared with uniformly isotope-labeled GB1 obtained without medium-switching and without the addition of the deuterated amino-acid mixture (39 ± 2 mg/L culture; see Fig. S3). It is likely that besides the addition of amino-acid mixture, switching the culture medium to a fresh one just before inducing the protein expression contributed to increasing the protein yield. Even for proteins with low expression levels, yield improvement can also be expected by applying this medium-switching method.

**Fig. 4:**
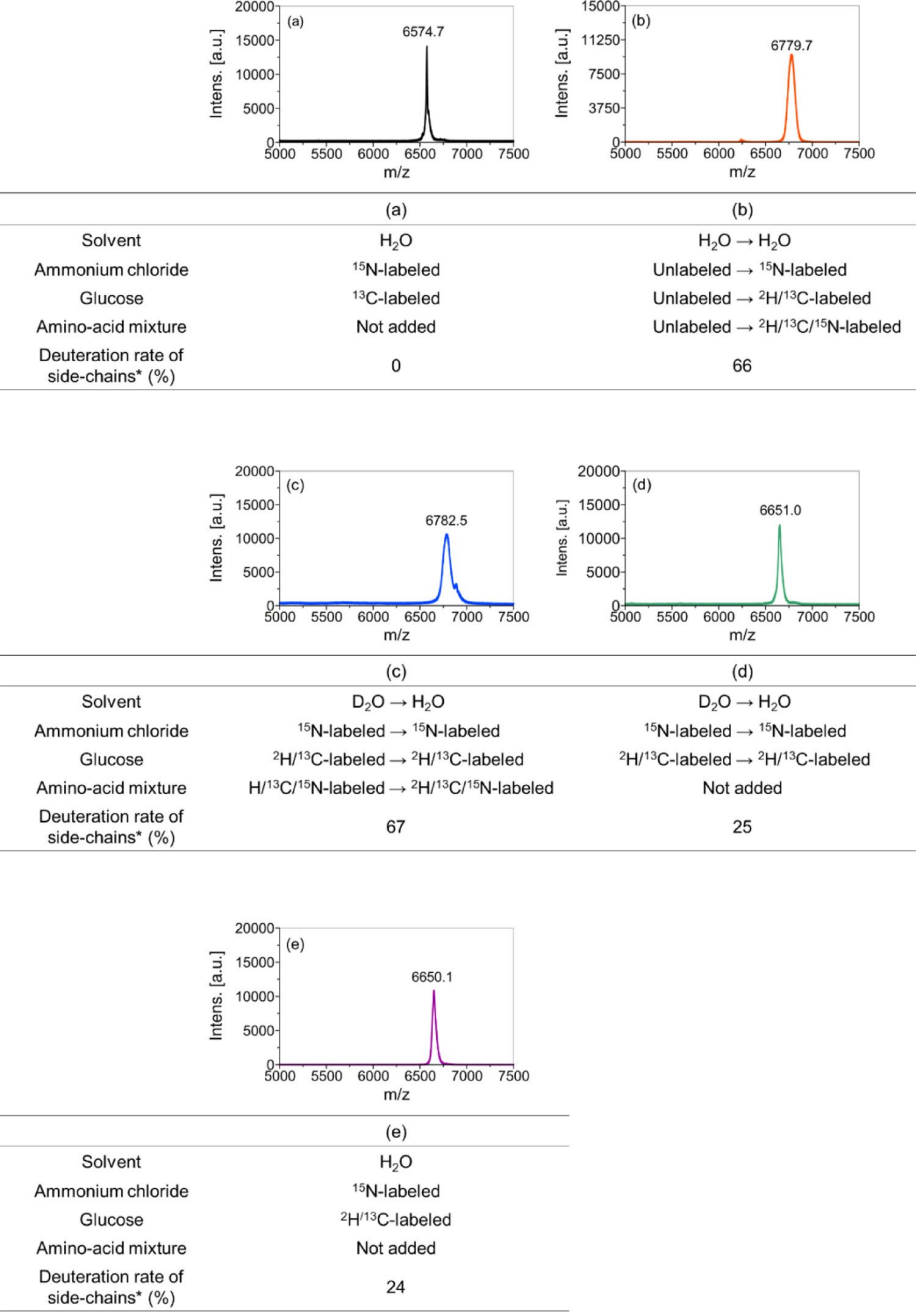
MALDI-TOF-MS results for isotope-labeled GB1 samples prepared (**b**, **c**, **d**) with and (**a**, **e**) without solvent/medium-switching, and (**b**, **c**) with and (**a**, **d**, **e**) without the addition of the deuterated amino-acid mixture. *The deuteration rate of side chains calculated from MALDI-TOF-MS results assumes that H^N^ and Hα are 100% protonated

Notably, the intensities of the side-chain peaks were suppressed or decreased, compared with those of the corresponding peaks for the fully protonated [^13^C, ^15^N]-labeled GB1, as indicated in Table 1. The results show that the aliphatic side chains of Ile, Leu, Phe, Trp, Tyr, and Val were almost completely deuterated with ^1^H intensities of 0–16%. Ala, Asp, Asn, Glu, Gln, Lys, and Thr showed notably attenuated side-chain relative intensity levels of 0−43%, except for Asp, Glu, Gln, and Met ^1^H_β_. Asp, Glu, and Gln showed considerably high relative ^1^H_β_ signal intensities at 74–79%, while Met ^1^H_β_ showed a signal intensity of ~ 100%. A separate analysis based on the mass spectroscopy (m/z = 6779.7, Fig. 4b) indicated ca. 66% of the side-chain deuteration level, which is consistent with a high deuteration level indicated by the NMR analysis. It should be noted that a similar analysis for a fully protonated sample indicated m/z = 6574.7 (Fig. 4a). We confirmed that the protein expression in H_2_O medium with ^15^N-labeled ammonium chloride, [^2^H, ^13^C]-labeled glucose, and [^2^H, ^13^C, ^15^N]-labeled amino-acid mixture without the medium-switching reduces the side-chain deuteration to 54% (Fig. S2c). Thus, the medium switching is necessary to optimize the deuteration level.

**Fig. 5:**
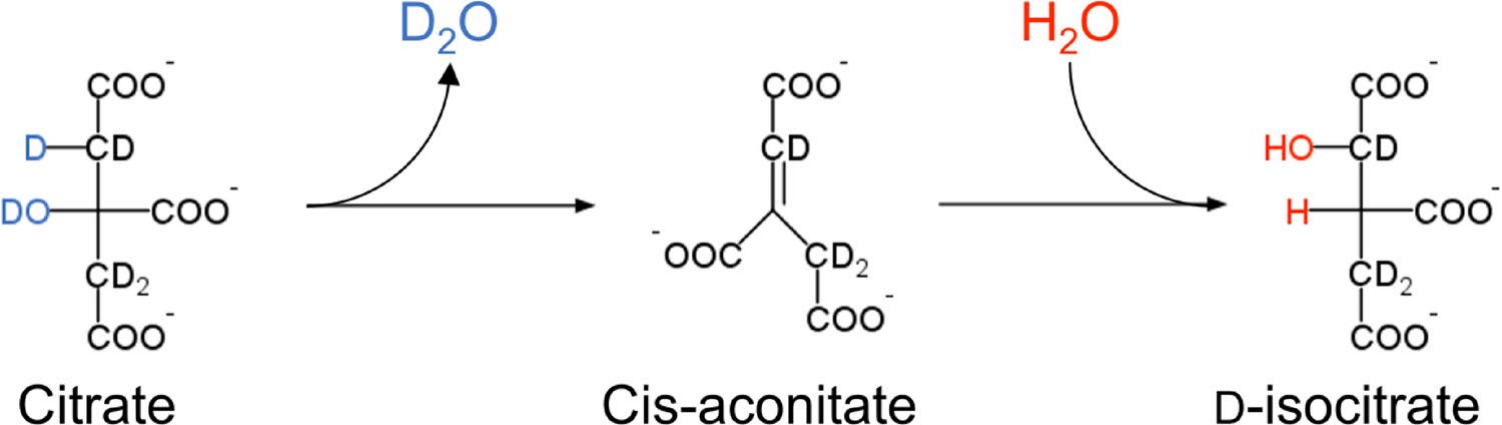
A reaction in the TCA cycle in which cis-aconitate is converted to D-isocitrate. In this reaction, protons derived from the solvent are incorporated into the skeleton from deuterated citrate

These results also suggest that the side-chain deuterium-labeling efficiency by this medium-switching method is amino-acid dependent. Amino acids such as Asp, Asn, Glu, and Gln with limited side-chain deuterium-labeling efficiency are known to be synthesized downstream in the TCA cycle (Maaheimo et al. 2001). The most compelling reasoning here is that the TCA cycle progressively produces back-protonated metabolic precursors such as oxaloacetate derived from deuterium-labeled glucose, incorporating protons from H_2_O into the side-chains of the amino acids that are synthesized downstream in the TCA cycle (Danmaliki et al. 2017). For example, the side-chain of the metabolic precursor corresponding to the β-position of Gln and Glu can be back-protonated when cis-aconitate is converted to D-isocitrate by aconitase via the incorporation of protons from the H_2_O solvent in the M9 media (Fig. 5).

**Fig. 6:**
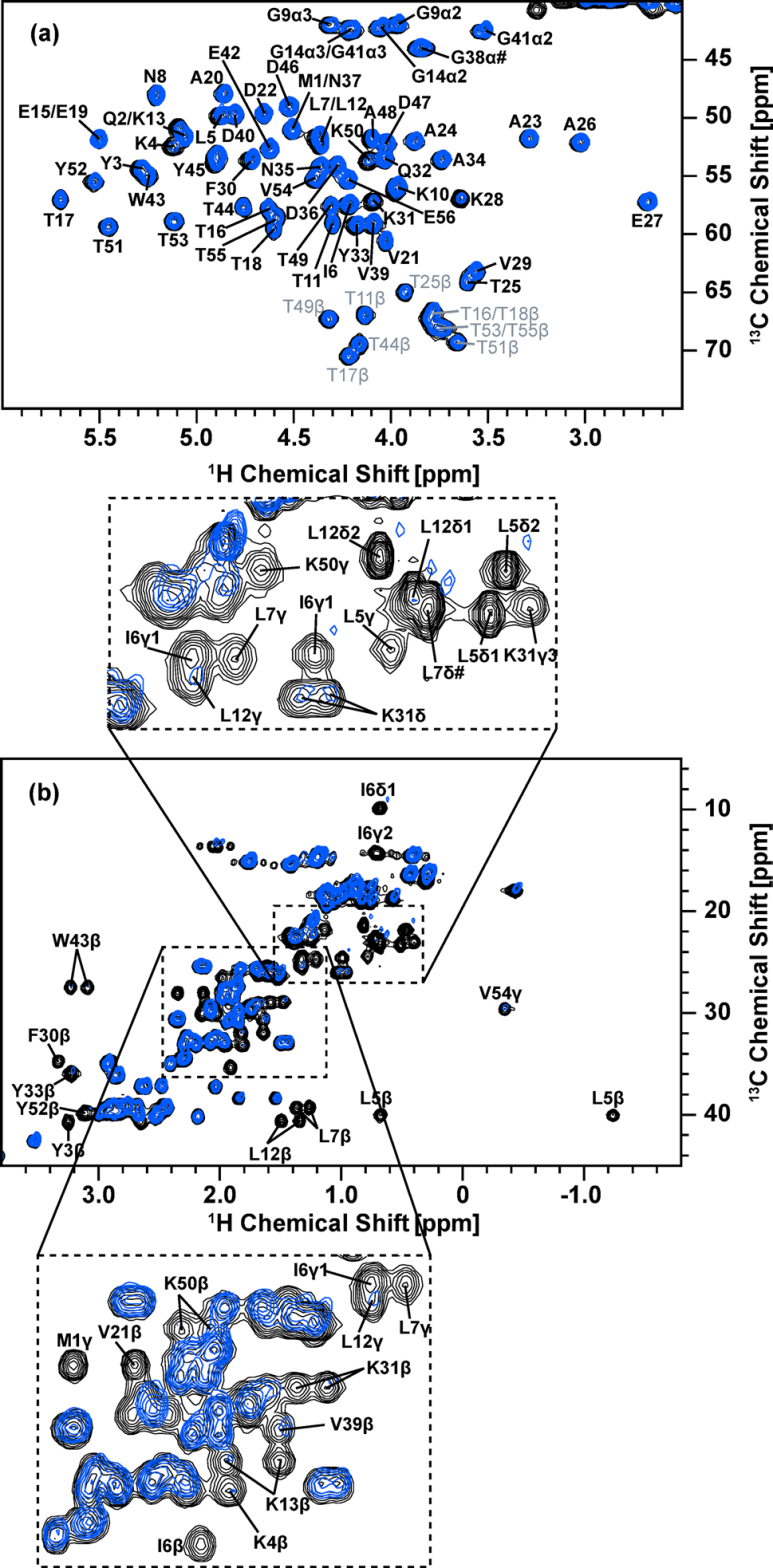
Overlay of ^1^H-^13^C HSQC solution NMR spectra of fully protonated GB1 (black) and selectively deuterated GB1 produced by the solvent-switching method with the deuterated amino-acid mixture (blue) in (**a**) ^1^H_α_/^13^C_α_ and (**b**) the side-chain aliphatic region. Both samples were dissolved in 50 mM phosphate buffer (pH 5.5), and these spectra were obtained with a 600 MHz solution NMR spectrometer equipped with a cryoprobe. The fully protonated and selectively deuterated GB1 protein concentrations were 83 and 92 µM, respectively. Contour levels are adjusted based on the protein concentration for each sample to compare signal intensities

To examine the effects of the solvent before the induction, we also produced isotope-labeled GB1 protein using a solvent-switching protocol from isotope-labeled D_2_O medium with ^15^N-labeled ammonium chloride, [^2^H, ^13^C]-labeled glucose, and [^2^H, ^13^C, ^15^N]-labeled amino-acid mixture to labeled H_2_O medium containing the same reagents. As indicated above, this solvent-switching protocol was our original idea to improve the side-chain deuteration efficiency. With this protocol, we could obtain GB1 protein at a comparable side-chain deuteration level (67%), based on the mass spectroscopy data (6782.5, Fig. 4c), to that for the medium-switching method (66%; with the deuterated amino-acid mixture) using an unlabeled amino-acid mixture before medium switching and an isotope-labeled amino-acid mixture after medium switching. Figure 6 shows a comparison of the 2D ^1^H-^13^C HSQC spectra of side-chain deuterated [^13^C, ^15^N]-labeled GB1 produced using the solvent-switching method with the [^2^H, ^13^C, ^15^N]-labeled amino-acid mixture (blue) and fully protonated [^13^C, ^15^N]-labeled GB1 (black) in (a) the ^1^H_α_/^13^C_α_ region and (b) the aliphatic side-chain region. For the deuterated [^13^C, ^15^N]-labeled GB1 sample, H_α_ atoms are maintained for all the residues. With the solvent-switching method, we observed stronger ^1^H_α_ signals for Lys, compared with those in Fig. 3a for the medium-switching method, for an unknown reason. In the aliphatic region, about half of the peaks observed in fully protonated [^13^C, ^15^N]-labeled GB1 are eliminated in the selectively deuterated sample (Fig. 6b). Overlaying the medium-switching and solvent-switching spectra, we can see that the signal patterns are quite similar (see Fig. S4). This solvent-switching method requires D_2_O and twice the amount of the isotope-labeled reagents, compared with the medium-switching method. We also produced the protein using twice the amount of [^2^H, ^13^C]-labeled glucose and [^2^H, ^13^C, ^15^N]-labeled amino-acid mixture to check the side-chain deuteration level by MALDI-TOF-MS (see Fig. S2d). The deuteration level was noticeably decreased (63%), compared with our proposed medium-switching (66%) and solvent-switching (67%) methods. Our proposed medium-switching method has achieved a higher side-chain deuteration level, while reducing the total cost for the isotope-labeled reagents and D_2_O.

Quantitative analysis by intensity comparison (excluding overlapped signals) showed that relative ^1^H_α_ signal intensities are ~ 78% on average, compared with those for the fully protonated GB1 sample. Thus, most of the ^2^H_α_ atoms are replaced by ^1^H_α_ in this protocol. Except for Ile, Lys, Thr, and α2 of Gly, for which relative ^1^H_α_ signal intensities to those of the fully protonated GB1 sample were respectively 61%, 36%, 49%, and 43%, all other amino acids showing resolved signals (see Table 2) indicate high levels of relative ^1^H_α_ signal intensities above 80%. In addition, the intensities of the side-chain peaks are suppressed or decreased, compared with those of the corresponding peaks for the fully protonated [^13^C, ^15^N]-labeled GB1. The results show that the aliphatic side chains of Ile, Leu, Phe, Trp, Tyr, and Val are almost completely deuterated (≤ 5%). While ^1^H side-chain signals were not fully suppressed, Ala, Asp, Asn, Glu, Gln, Lys, and Thr show reasonable attenuation for side-chain ^1^H_β_ and ^1^H_γ_ signals at 0−63% and 0–34%, respectively, except for Met ^1^H_β_, which was barely replaced by ^2^H. Those results are comparable to those of the medium-switching protocol from H_2_O to H_2_O. While twice the amounts of isotope-labeled materials are needed, the yield (77 ± 13 mg/L culture) for this protocol was improved, compared with that for the medium-switching protocol (58 ± 3 mg/L). Thus, this method has its merits with mildly improved side-chain deuteration (see Table 2).

**Table 2:** Relative peak intensities for the aliphatic region in a 2D ^1^H-^13^C HSQC solution NMR spectrum of GB1 prepared by solvent-switching from D_2_O medium containing the deuterated amino-acid mixture to H_2_O medium containing the deuterated amino-acid mixture

Amino-acid type	Normalized signal intensities attributed to a chemical group (%)				
	α	β	γ	δ	ε
Alanine	86.2 ± 1.3	36.3 ± 0.6			
Asparagine	80.7 ± 2.4	44.7 ± 2.2			
Aspartate	103.5 ± 1.9	61.2 ± 2.2			
Glutamate	95.4 ± 1.6	63.4 ± 1.5	34.3 ± 1.0		
Glutamine	82.1 ± 3.4	54.3 ± 3.2	29.1 ± 1.6		
Glycine	α2: 43.4 ± 1.9 α3: 96.3 ± 2.7				
Isoleucine	60.8 ± 2.9	0	γ1: 0 γ2: 0	0	
Leucine	84.4 ± 2.4	0	0	0	
Lysine	36.1 ± 1.1	0	0	6.4 ± 0.3	25.3 ± 0.7
Methionine^1)^	*	97.4 ± 8.3	0	-	0
Phenylalanine	84.8 ± 4.4	0			
Threonine	49.2 ± 0.9	35.4 ± 0.7	28.3 ± 0.5		
Tryptophan^1)^	*	0			
Tyrosine	84.4 ± 2.4	0			
Valine	100.5 ± 1.8	0	5.2 ± 0.2		

1) For these amino acids, signals indicated by * could not be measured due to signal overlap with other peaks

The intensities listed for each amino-acid type are normalized by the corresponding intensities of fully protonated GB1. To adjust differences in the sample concentrations, the peak intensities of the selectively deuterated sample used in the calculations are normalized by a scaling factor, which is the concentration of the selectively deuterated sample divided by the concentration of the fully protonated sample. In cases where multiple residues having the same amino-acid type are present, the average values are indicated. Errors were calculated by error propagation with noise

**Fig. 7:**
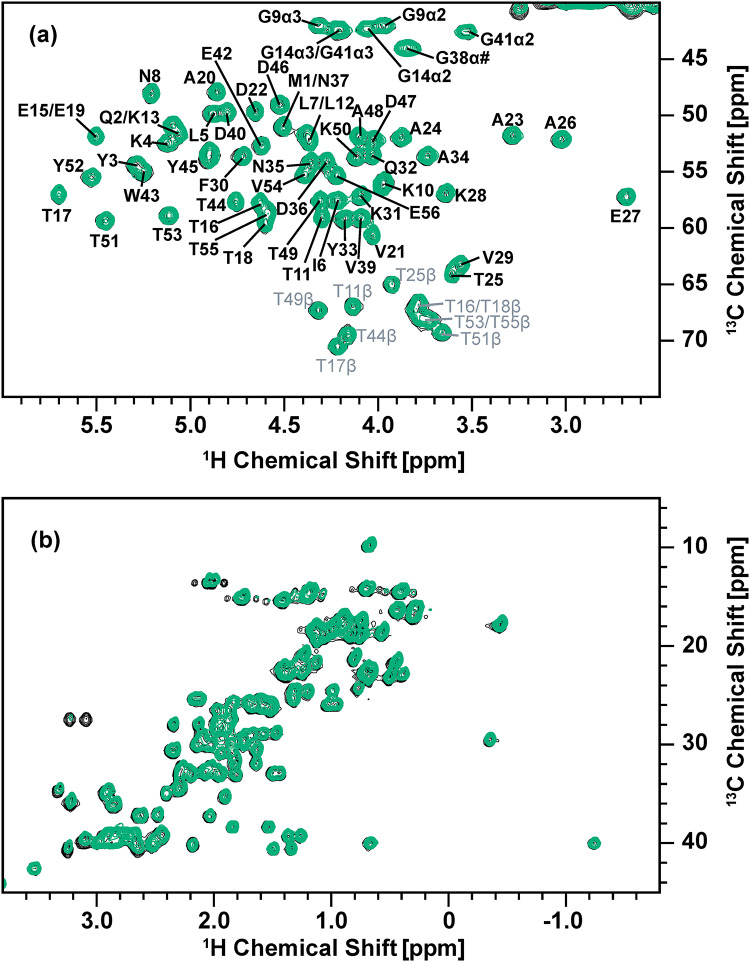
Overlay of ^1^H-^13^C HSQC solution NMR spectra of fully protonated GB1 (black) and selectively deuterated GB1 produced by the solvent-switching method without the deuterated amino-acid mixture (green) in (**a**) ^1^H_α_/^13^C_α_ and (**b**) the aliphatic regions. Both samples were dissolved in 50 mM phosphate buffer (pH 5.5), and these spectra were obtained with a 600 MHz solution NMR spectrometer equipped with a cryoprobe. The fully protonated and selectively deuterated GB1 protein concentrations were 83 and 77 µM, respectively. Contour levels are adjusted based on the protein concentration for each sample to compare signal intensities

To confirm the effect of the deuterated amino-acid mixture in the H_2_O M9 medium, we also performed the corresponding experiments for deuterated [^13^C, ^15^N]-labeled GB1 produced by the solvent exchange method without [^2^H, ^13^C, ^15^N]-labeled amino-acid mixture in the medium. Again, H_α_ atoms are back-exchanged from ^2^H to ^1^H in most amino-acid types (Fig. 7a). However, the spectral analysis of the aliphatic side-chain region suggests a high level of protonation; almost all of the ^1^H/^13^C correlation peaks except for the β-position of tryptophan remain (Fig. 7b). Furthermore, quantitative analysis of the side-chain signals (Table 3) also showed that the side-chain ^1^H_β_ signals exhibit high relative intensities at ~ 80% while other side-chain ^1^H species exhibit relative intensities at 50–87%, except for Met ^1^H_ε_ (26%). Mass spectrometry results (6651.0; estimated side-chain deuteration 25%) in Fig. 4d also indicate a low deuteration level, which is consistent with the NMR analysis results. Thus, employing the deuterated amino-acid mixtures is mandatory to prepare target proteins with higher rates of deuteration at 65% or higher, as shown in Fig. 4b, c.

**Table 3:** Relative peak intensities for the aliphatic region in a 2D ^1^H-^13^ C HSQC solution NMR spectrum of GB1 that was produced by the solvent-switching method without the deuterated amino-acid mixture

Amino-acid type	Normalized signal intensities attributed to a chemical group (%)				
	α	β	γ	δ	ε
Alanine	111.8 ± 1.6	57.0 ± 0.7			
Asparagine	75.2 ± 2.6	68.7 ± 2.8			
Aspartate	122.0 ± 2.3	73.1 ± 2.7			
Glutamate	108.8 ± 2.7	96.7 ± 1.8	61.0 ± 1.7		
Glutamine	108.0 ± 4.6	102.6 ± 3.3	58.5 ± 2.2		
Glycine	α2: 90.6 ± 2.8 α3: 109.4 ± 3.9				
Isoleucine	94.6 ± 3.9	119.3 ± 8.5	γ1: 78.1 ± 4.2 γ2: 34.3 ± 1.4	49.6 ± 0.7	
Leucine	96.9 ± 2.9	88.6 ± 3.9	85.8 ± 2.8	86.5 ± 2.3	
Lysine	105.5 ± 1.8	78.7 ± 2.4	66.6 ± 2.4	73.1 ± 1.2	92.5 ± 1.0
Methionine^1)^	*	138.4 ± 11.1	63.8 ± 2.4	-	26.1 ± 0.5
Phenylalanine	109.9 ± 5.5	122.9 ± 15.8			
Threonine	102.0 ± 1.3	70.6 ± 1.1	70.7 ± 0.7		
Tryptophan^1)^	*	0			
Tyrosine	132.7 ± 3.5	56.6 ± 2.6			
Valine	99.6 ± 1.9	78.9 ± 3.1	45.0 ± 0.5		

1) For these amino acids, signals indicated by * could not be measured due to signal overlap with other peaks

The intensities listed for each amino-acid type are normalized by the corresponding intensities of fully protonated GB1. To adjust differences in the sample concentrations, the peak intensities of the deuterated sample used in the calculations are normalized by a scaling factor, which is the concentration of the deuterated sample divided by the concentration of the fully protonated sample. In cases where multiple residues having the same amino-acid type are present, the average values are indicated. Errors were calculated by error propagation with noise

It should be noted that the observed protonation levels for different amino-acid types are consistent with those obtained with an inverse fractional deuteration method in a previous study by Medeiros-Silva et al. (Medeiros-Silva et al. 2016), in which they produced ubiquitin in a similar H_2_O medium (^15^N-labeled ammonium chloride and [^2^H, ^13^C]-labeled glucose) without any solvent/medium-switching. We prepared GB1 protein in an H_2_O medium containing 0.5 g/L ^15^N-labeled ammonium chloride and 2.0 g/L [^2^H, ^13^C]-labeled glucose as described in their work. The mass spectrometry analysis (m/z = 6650.1; Fig. 4e) indicates the side-chain-deuteration level at 24%, which is also substantially lower than that in the medium-switching method (66%). This deuteration level is almost the same (6651.0; estimated side-chain deuteration 24%) as that of GB1 prepared with the medium composition after solvent-switching of our sample without the deuterated amino-acid mixture (see Fig. S5 about the solution NMR results, Fig. S6 about SSNMR results, and Table S2 about line width comparison based on the SSNMR results). We concluded that the deuteration efficiency can be notably improved by the medium/solvent switching methods with the deuterated amino-acid mixture, compared with that of the fractional deuteration method (Medeiros-Silva etal. 2016).

### Resolution improvement by the solvent-switching method

**Fig. 8:**
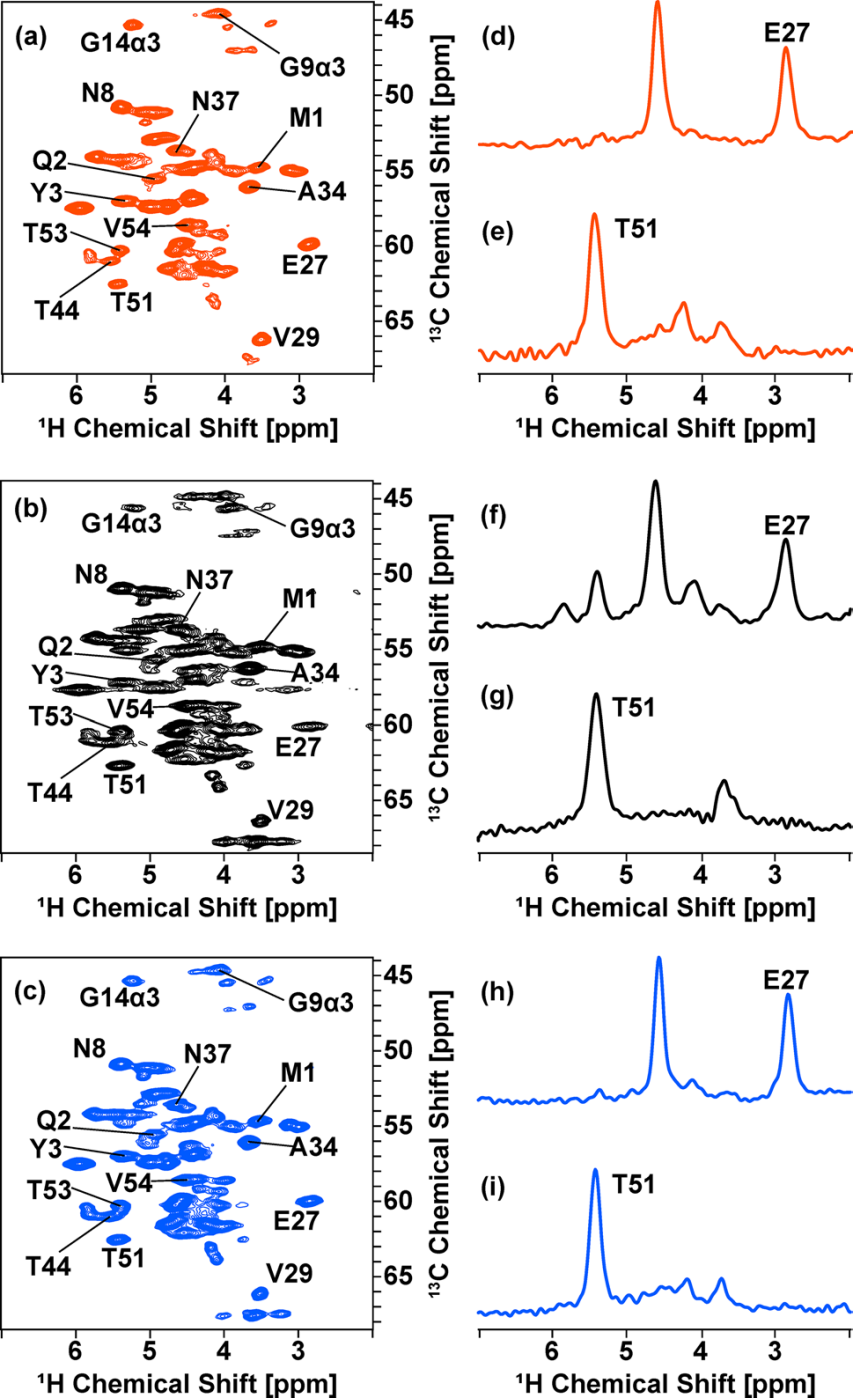
Magnified ^1^H_α_/^13^C_α_ regions in the ^1^H-detected 2D ^1^H/^13^C correlation SSNMR spectra of (**a**) a side-chain deuterated microcrystalline GB1 sample produced with the unlabeled before switching the medium and with the deuterated amino-acid mixture after switching the medium, (**b**) a fully protonated microcrystalline GB1 sample, and (**c**) a side-chain-deuterated microcrystalline GB1 sample produced by the solvent-switching method with the deuterated amino-acid mixture. (**d**−**i**) Comparison of 1D ^1^H slices selected at the positions of Glu 27 (^13^C chemical shift of 59.967 ppm; **d**, **f**, **h**) and Thr 51 (^13^C chemical shift of 62.571 ppm; **e**, **g**, **i**) for the same GB1 samples (**d**, **e**) selectively deuterated with the medium-switching method and addition of unlabeled amino-acid mixture before switching medium and the deuterated amino-acid mixture after switching the medium (**f**, **g**) fully protonated, and (**h**, **i**) selectively deuterated with the deuterated amino-acid mixture and the solvent-switching method. The spectra were obtained using a 900 MHz JEOL spectrometer with 70 kHz MAS. The 1D and 2D data were processed by NMRPipe/NMRDraw software

Next, we examined improvements in ^1^H SSNMR resolution for samples prepared by selective deuteration with the medium- and solvent-switching methods. Figure 8 compares the ^1^H-detected 2D ^1^H/^13^C correlation SSNMR spectra of (a, c) selectively deuterated microcrystalline [^13^C, ^15^N]-labeled GB1 samples prepared with (a) medium-switching method with the unlabeled amino-acid mixture before switching the medium and [^2^H, ^13^C, ^15^N]-labeled amino-acid mixture after switching the medium (red) and (c) solvent-switching method with [^2^H, ^13^C, ^15^N]-labeled amino-acid mixture (blue) with a corresponding 2D SSNMR spectrum of (b) a fully protonated [^13^C, ^15^N]-labeled GB1 microcrystalline sample (black). The data were obtained with ultra-fast MAS at a spinning rate of 70 kHz. The spectra in Fig. 8(a–c) for the three samples showed 13 resolved peaks in 60 peaks amenable to quantitative analysis of ^1^H line widths (Table 4). The line widths of these 13 peaks were compared with the corresponding line widths of the side-chain-selectively deuterated sample to evaluate spectral resolution (see 1D slices in Fig. 8d–i). It was confirmed that, for almost all the resolved peaks, the ^1^H line widths of the selectively deuterated sample produced with the deuterated amino-acid mixture are narrower than those of the fully protonated sample, as shown in Fig. 8(d, e) (see Fig. S7 for full assignments of the ^1^H_α_/^13^C_α_ region spectrum). The resolution improvement factors are in the range of (a) 0.92–1.94 and (c) 1.03–1.65 over the control data in (a); the average improvement factor is (a) 1.21 and (c) 1.22 (see Table 4). For Thr 51, the line width for the fully protonated sample is 166 Hz (Fig. 8f). In comparison, that of the solvent-switched deuterated sample is 156 Hz (Fig. 8j), resulting in an average resolution improvement by a factor of ~ 1.07. In some other residues, medium- and solvent-switching methods enable us to improve ^1^H line widths dramatically. For example, for Glu 27, the ^1^H line widths are (a) 131 Hz, (b) 251 Hz, and (c) 152 Hz (see also slices in (d, f, h)), resulting in significant resolution improvement by a factor of (a) 1.94 and (c) 1.65. Line width comparisons for other isolated peaks are described in Table 4 for the samples prepared with and without the [^2^H, ^13^C, ^15^N]-labeled amino-acid mixture, respectively. These results show that notable resolution enhancement can be achieved by the medium- and solvent-switching methods and supplementation with a suitable level of [^2^H, ^13^C, ^15^N]-labeled amino-acid mixture in the medium. For the inverse fractional deuterated sample, the resolution was mildly improved by a factor of 1.12 (data not shown). Thus, there is a notable advantage for the medium- and solvent-switching methods.

**Table 4:** ^1^H line widths of side-chain-deuterated GB1 samples produced by the medium-switching method (with the addition of the unlabeled and deuterated amino-acid mixture in the medium before and after induction) and by the solvent-switching method (with the addition of the deuterated amino-acid mixture) and their resolution enhancement factors compared with a fully protonated GB1 sample

Residue number	Fully protonated (Hz)	Medium-switching deuteration with the amino-acid mixture (Hz) (Resolution enhancement factor)	Solvent-switching deuteration with the amino-acid mixture (Hz) (Resolution enhancement factor)
M1	144	133 (1.08)	132 (1.09)
Q2	190	175 (1.08)	167 (1.14)
Y3	228	220 (1.04)	159 (1.44)
N8	157	139 (1.13)	142 (1.10)
G14α3	182	140 (1.30)	176 (1.03)
E27	251	129 (1.94)	152 (1.65)
V29	131	142 (0.92)	120 (1.09)
A34	138	109 (1.27)	111 (1.24)
N37	158	129 (1.23)	136 (1.16)
T44	173	144 (1.20)	135 (1.28)
T51	166	156 (1.07)	145 (1.15)
T53	186	149 (1.25)	143 (1.30)
V54	163	132 (1.23)	138 (1.18)
		Resolution enhancement average: 1.21	Resolution enhancement average: 1.22

^1^H line widths were determined from well-dispersed peaks in the ^1^H_α_/^13^C_α_ region of ^1^H-detected 2D ^1^H/^13^C correlation SSNMR spectra (Fig. 8). The resolution enhancement factor for a selectively deuterated sample was defined by a ratio of the line width of a fully protonated sample to that of the selectively deuterated sample for each residue

For some amino acids, we observed higher suppression of side-chain ^1^H signals with a larger amount of amino-acid mixture (2.0 g/L; see Fig. S8 and S9). However, 1.0 g/L of the amino-acid mixture used for Fig. 3 was found to be sufficient to suppress side-chain ^1^H signals for most amino-acids (Fig. S10). Thus, we believe that the addition of 1.0 g/L of the amino-acid mixture is a recommended level for most applications of the medium-/solvent-switching method. Further optimization for a specific application is possible by changing the amino-acid composition of the amino-acid mixture or adding amino-acids with different isotope-labeling schemes.

### Stereospecific assignment for Glycine residues

Based on solution NMR data, the ratio of the ^1^H signal intensity of α3 of glycine to that of α2 was estimated at ~ 2.0 with the medium-/solvent-switching methods as shown in Tables 1 and 2. The results are consistent with notable simplification of the SSNMR　spectra in Gly region in Fig. 8(a, c). These results suggested that the α3 position in glycine was preferably protonated. Our method produced the target protein in H_2_O with a deuterated amino acid mixture and deuterated glucose. Loscha and Otting reported that the α3 position of protonated glycine was selectively deuterated by serine hydroxymethyltransferase by expressing the protein in D_2_O using protonated glycine (Loscha and Otting 2013). Glycine can be produced by serine hydroxymethyltransferase. Based on the reports by Movellan et al. (Movellan et al. 2019), we speculate that this reaction may involve transaminase and possibly other additional metabolic pathways. In any case, our method is likely to be useful for stereoselective Gly assignments.

## Conclusion

We successfully demonstrated a simple yet effective method for side-chain-selective deuteration using the model protein GB1 by switching the cell-culture media just before induction with a deuterated amino-acid mixture supplemented to the media. The aliphatic side chains of Ile, Leu, Phe, Tyr, Trp, and Val are completely deuterated, while those of other amino acids except for Met H_β_ are partially deuterated at a reasonably high level. Furthermore, the α-position is protonated at a high level due to back-protonation derived from the solvent after induction. Our results clearly demonstrated that this simple method offers an excellent means to improve ^1^H spectral resolution as well as to simplify side-chain signals for structural studies by ^1^H-detected protein SSNMR. This method also enables us to easily assign backbone signals of proteins by improving ^1^H resolution, including those for stereo-selectively isotope-labeled Gly residues. Residual ^1^H side-chain signals may be useful for collecting distance constraints without excess signal overlaps. More complete side-chain-signal suppression and further ^1^H resolution improvement of ^1^H_α_ signals may be possible by adding TCA-cycle inhibitors and/or optimizing the levels of isotope-labeled amino-acids or precursors. We also confirmed that the yields of the selectively labeled protein by our methods are much higher than those obtained by conventional methods for expressing a fully protonated protein. Although we used a commercial amino-acid mixture prepared from algal lysate for cost-effectiveness and convenience, the level of each deuterated amino-acid in the medium can be tailored by adding chemically synthesized amino-acids if needed. In terms of cost-effectiveness, our medium-switching method is an excellent choice. Selective deuteration based on the medium-/solvent-switching methods described here will contribute to further developments of SSNMR-based structural analysis of complex proteins such as amyloid fibrils and proteins bound to cell membranes.

## Supplementary Information

Below is the link to the electronic supplementary material.

Supplementary Material 1

## Data Availability

No datasets were generated or analysed during the current study.
