# Supplementary material for: Side-chain-selective deuterium labeling by a simple bio-expression method enhances resolution and simplifies spectra in 1H-detected protein solid-state NMR
Source: J Biomol NMR. 2025 Nov 1;79(4):315–30. doi: 10.1007/s10858-025-00476-9 (PMC12664835; doi:10.1007/s10858-025-00476-9)
Supplement: Supplementary file 1 — Supplementary Material 1 [file 10858_2025_476_MOESM1_ESM.pdf]

Supporting Information for "Side-chain-selective Deuterium Labeling by a Simple Bio-expression Method Enhances Resolution and Simplifies Spectra in ^{1}H-detected Protein Solid-state NMR" by Yoshiki Shigemitsu, Yuki Miyazaki, Hibiki Terami, Daria Ostapova, Tatsuya Matsunaga, Ryo Takahashi, Takumi Inoue, Toshio Yamazaki and, Yoshitaka Ishii

The information contains supporting Tables (Tables S1–2) and supporting data (Figures S1–10).

Trace element (filtration for sterilization)	Amount in 100 mL
$CaCl_2 \cdot 2H_2O$	600 mg
$CoCl_2 \cdot 6H_2O$	80 mg
$CuCl_2 \cdot 2H_2O$	30 mg
$FeSO_4 \cdot 7H_2O$	600 mg
H_3BO_3	2 mg
$MnCl_2 \cdot 4H_2O$	115 mg
$(NH_4)_6MO_7O_{24} \cdot 4H_2O$	25 mg
$ZnSO_4 \cdot 7H_2O$	70 mg
Disodium EDTA$\cdot 2H_2O$	500 mg

Table S1. Concentrations of trace elements used for cell expression.

Residue number	Fully protonated (Hz)	*Inverse*-fractional deuteration (Hz) (Resolution enhancement)	*Solvent*-switching deuteration *without* the amino-acid mixture (Hz) (Resolution enhancement)
M1	144	128 (1.12)	118 (1.22)
Q2	190	215 (0.88)	206 (0.92)
Y3	228	185 (1.23)	192 (1.19)
N8	157	142 (1.10)	161 (0.98)
G14α3	182	232 (0.78)	180 (1.01)
E27	251	177 (1.42)	161 (1.56)
V29	131	116 (1.13)	121 (1.08)
A34	138	125 (1.10)	129 (1.07)
N37	158	137 (1.15)	156 (1.02)
T44	173	151 (1.15)	157 (1.10)
T51	166	157 (1.06)	160 (1.04)
T53	186	151 (1.23)	146 (1.28)
V54	163	154 (1.06)	145 (1.12)
		Resolution enhancement average: 1.11	Resolution enhancement average: 1.12

Table S2. Comparison of ^{1}H line widths in SSNMR among fully protonated GB1 and side-chain-deuterated GB1 produced by the inverse fractional deuteration method and by the solvent-switching method (without the addition of the deuterated amino-acid mixture), respectively. ^{1}H line widths determined from well-dispersed peaks in the $^{1}H_{\alpha}/^{13}C_{\alpha}$ region of ^{1}H-detected 2D $^{1}H/^{13}C$ correlation SSNMR spectra (Fig. S6)

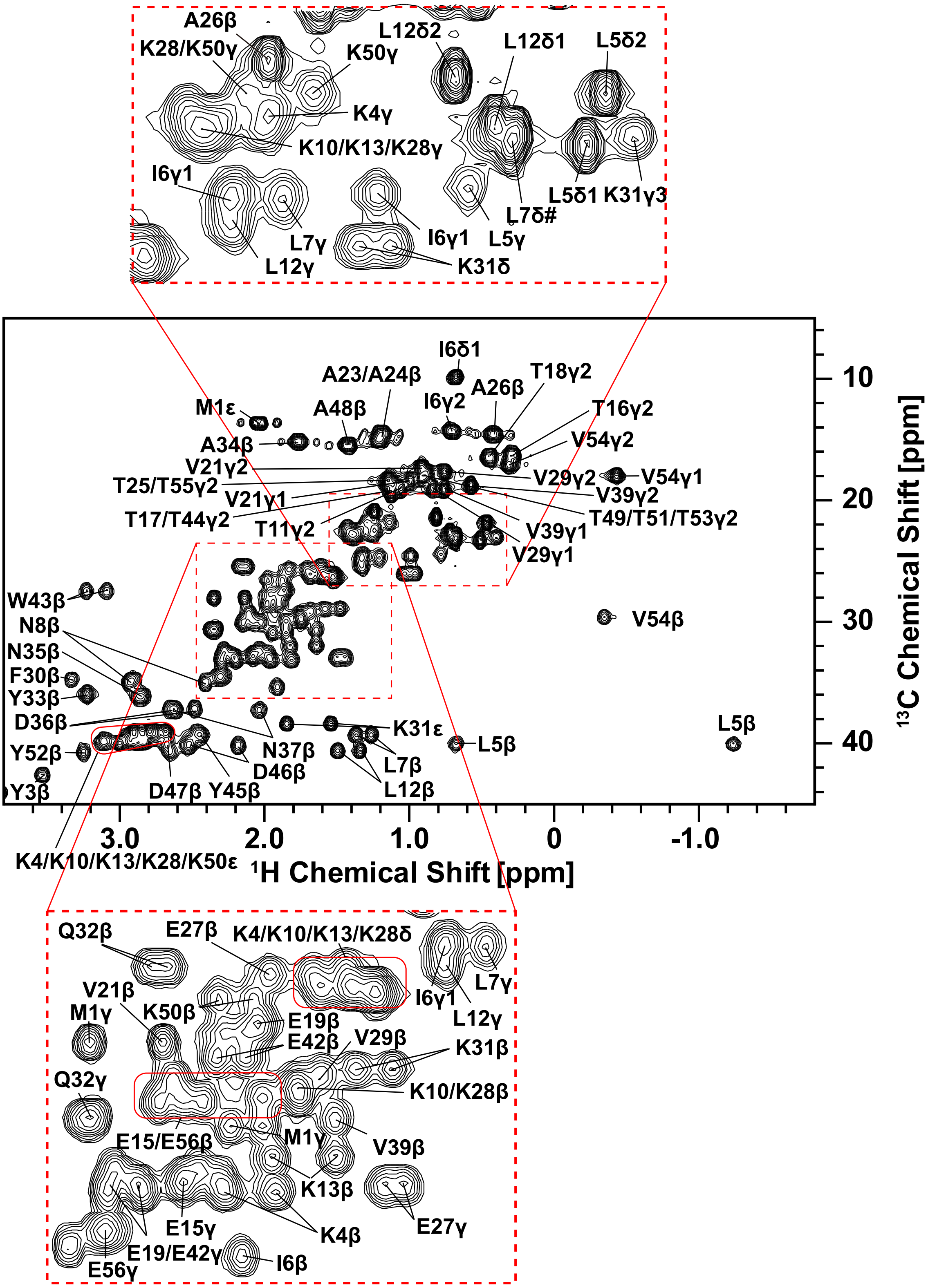

Figure S1. Side-chain aliphatic region of the ^{1}H-^{13}C HSQC spectrum of fully protonated GB1. The data, which are the same as those in Fig. 3, are displayed with signal assignments.

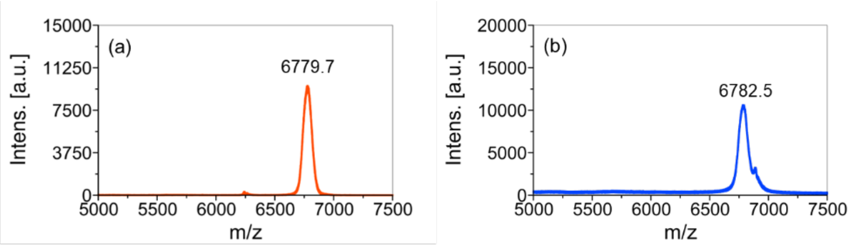

	(a)	(b)
Solvent	$H_2O \rightarrow H_2O$	$D_2O \rightarrow H_2O$
Ammonium chloride	^{15}N-labeled → ^{15}N-labeled	Unlabeled → ^{15}N-labeled
Glucose	^{2}H/^{13}C-labeled → ^{2}H/^{13}C-labeled	Unlabeled → ^{2}H/^{13}C-labeled
Amino-acid mixture	^{2}H/^{13}C/^{15}N-labeled → ^{2}H/^{13}C/^{15}N-labeled	Unlabeled → ^{2}H/^{13}C/^{15}N-labeled
Deuteration rate of side-chains* (%)	66	67

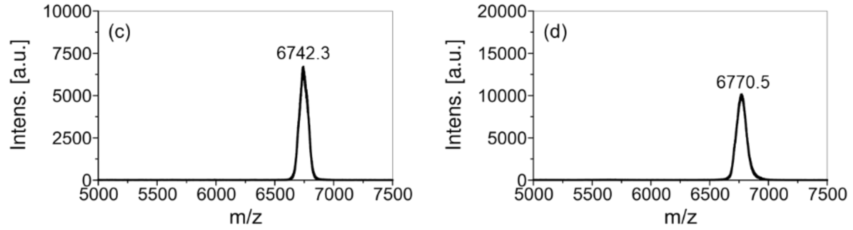

	(c)	(d)
Solvent	H_2O	H_2O
Ammonium chloride	^{15}N-labeled	^{15}N-labeled
Glucose	^{2}H/^{13}C-labeled	^{2}H/^{13}C-labeled (Double the amount)
Amino-acid mixture	^{2}H/^{13}C/^{15}N-labed	^{2}H/^{13}C/^{15}N-labeled (Double the amount)
Deuteration rate of side-chains* (%)	54	63

Figure S2. MALDI-TOF-MS results for isotope-labeled GB1 samples prepared (a) with medium-switching from H_2O to H_2O with the addition of the deuterated amino-acid mixture (same as Fig. 4b), (b) with solvent-switching from D_2O with the unlabeled amino-acid mixture to H_2O with the deuterated amino-acid mixture (same as Fig. 4c), (c) with the addition of the deuterated amino-acid mixture but without solvent- or medium-switching, and (d) with twice the amount of glucose, and the deuterated amino-acid mixture without the medium-switching. *The deuteration rate of side-chains calculated from MALDI-TOF-MS results assumes that H^N and Hα are 100% protonated.

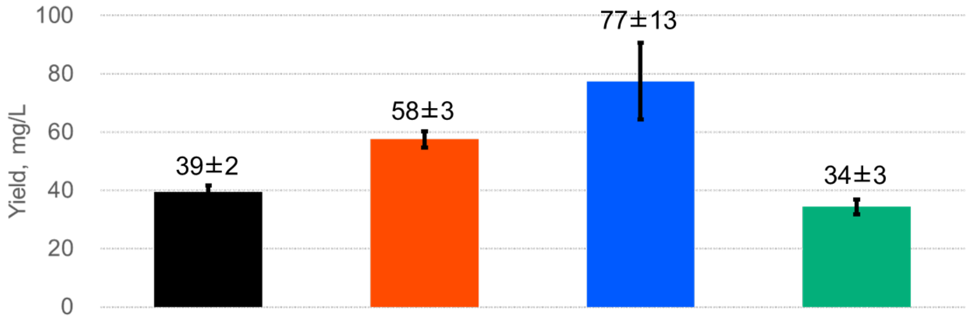

	(a)	(b)	(c)	(d)
Solvent	H_2O	$H_2O \rightarrow H_2O$	$D_2O \rightarrow H_2O$	$D_2O \rightarrow H_2O$
Ammonium chloride	^{15}N-labeled	unlabeled $\rightarrow$ ^{15}N-labeled	^{15}N-labeled $\rightarrow$ ^{15}N-labeled	^{15}N-labeled $\rightarrow$ ^{15}N-labeled
Glucose	^{13}C-labeled	unlabeled $\rightarrow$ ^{2}H/^{13}C-labeled	^{2}H/^{13}C-labeled $\rightarrow$ ^{2}H/^{13}C-labeled	^{2}H/^{13}C-labeled $\rightarrow$ ^{2}H/^{13}C-labeled
Amino-acid mixture	Not added	unlabeled $\rightarrow$ ^{2}H/^{13}C/^{15}N-labeled	^{2}H/^{13}C/^{15}N-labeled $\rightarrow$ ^{2}H/^{13}C/^{15}N-labeled	Not added

Figure S3. Yield comparison for isotope-labeled GB1 samples prepared (b, c) with and (a, d) without solvent/medium-switching, and (b, c) with and (a, d) without the addition of the deuterated amino-acid mixture. When comparing the yields of proteins, we found that there were variations in yield depending on the reagent lot of the phosphate buffer. Therefore, for this experiment, we added 1 mL of the trace elements listed in Table S1 per 1 L of M9 media to promote growth in place of 0.1 mM $CaCl_2$ in the materials and methods.

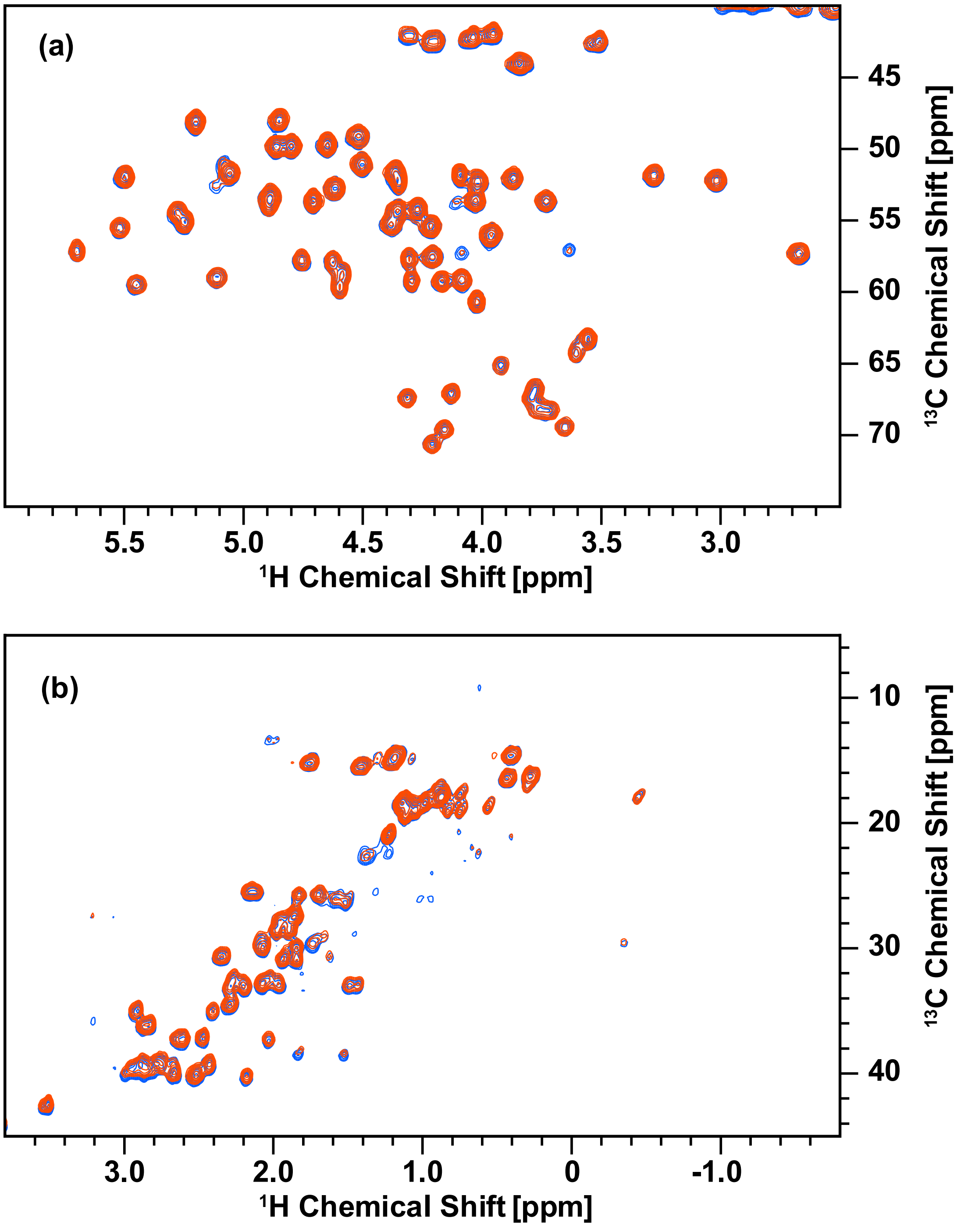

Figure S4. Overlay of ^{1}H-^{13}C HSQC solution NMR spectra of selectively side-chain deuterated GB1 produced by the medium-switching method with the unlabeled amino-acid mixture in the medium before switching the medium and with the deuterated amino-acid mixture after switching the medium (red) and by the solvent-switching method with the deuterated amino-acid mixture (blue) in (a) $^{1}H_{\alpha}/^{13}C_{\alpha}$ and (b) the side-chain aliphatic region. Both samples were dissolved in 50 mM phosphate buffer (pH 5.5), and these

spectra were obtained with a 600 MHz solution NMR spectrometer equipped with a cryoprobe. The GB1 concentrations of medium-switching and solvent-switching were 92 and 96 μM, respectively. Contour levels are adjusted based on the protein concentration for each sample.

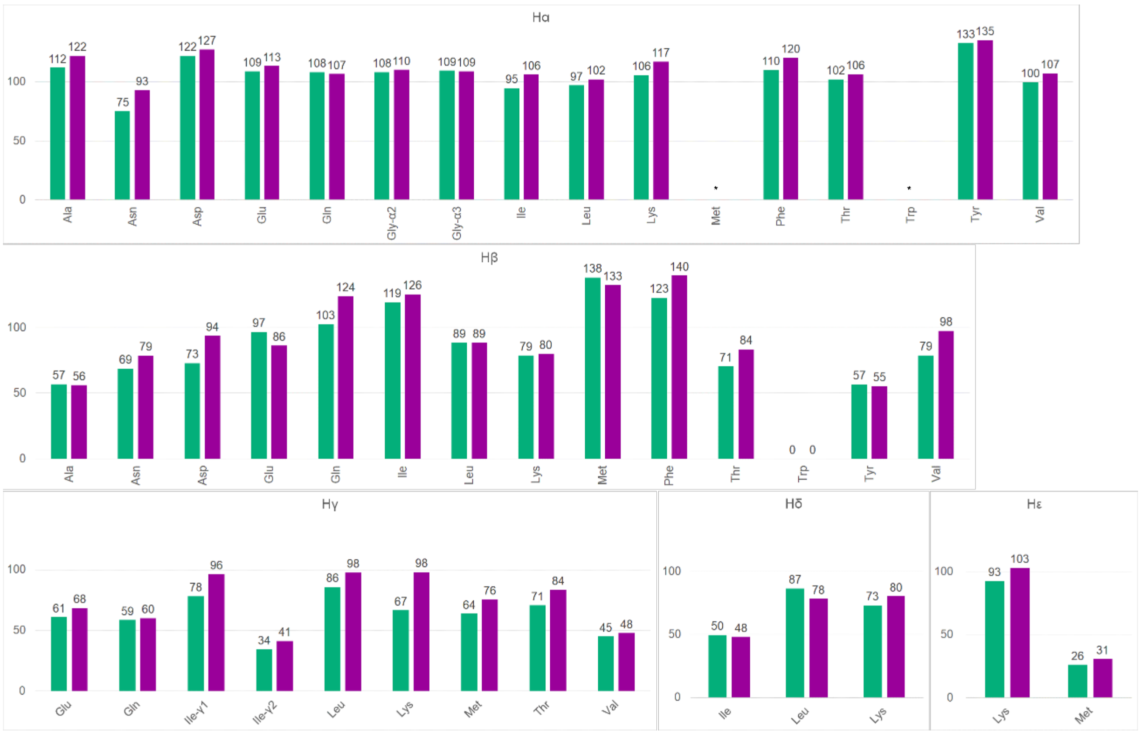

Figure S5. Relative-intensity comparison of side-chain peaks of GB1 proteins by solution NMR. Green and purple bars show the result of GB1 produced by the solvent-switching method without the deuterated amino-acid mixture and by the inverse fractional deuteration method, respectively. The signals * could not be measured due to signal overlap with other peaks.

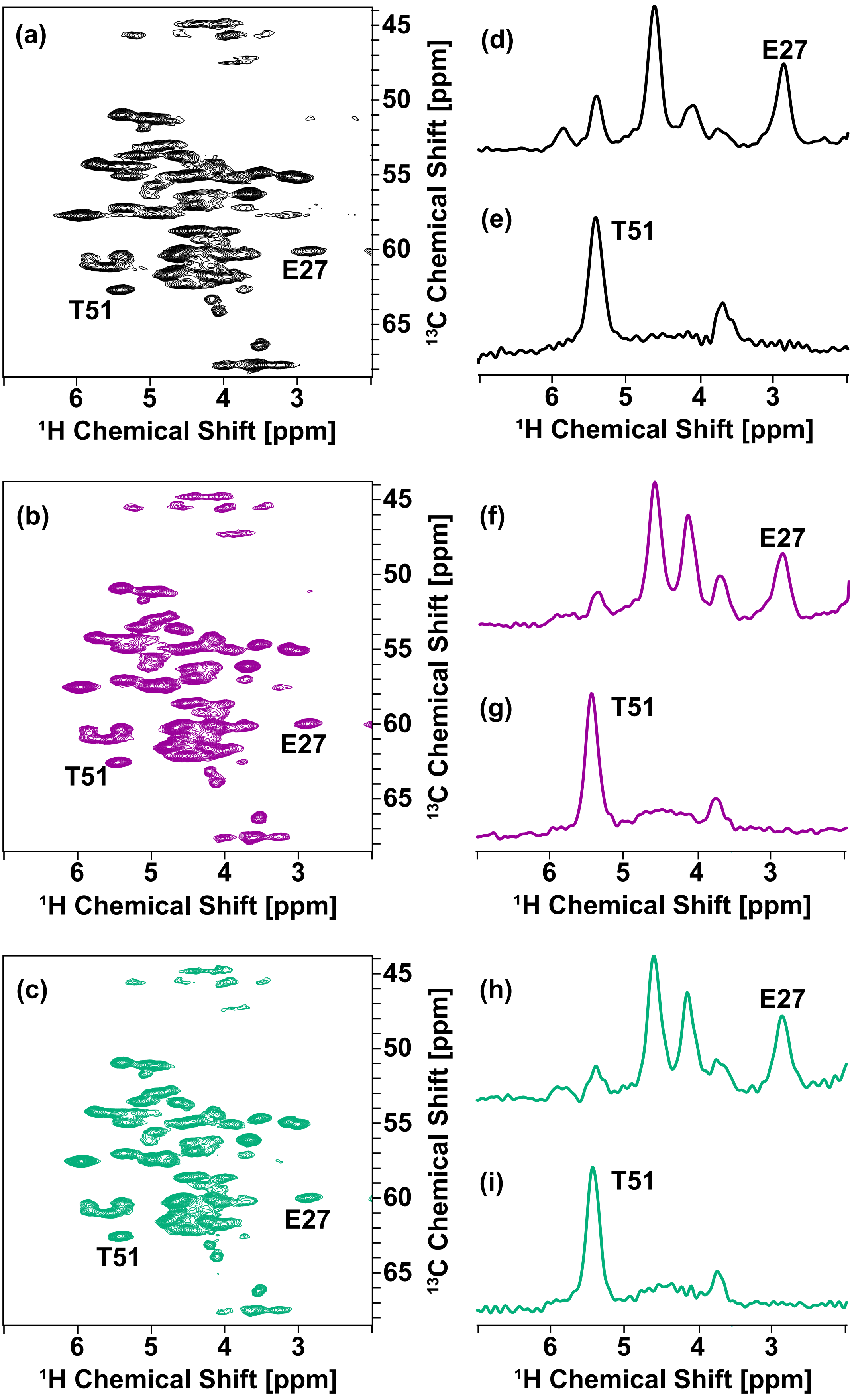

(a)
(b)
(c)
(d)
(e)
(f)
(g)
(h)
(i)
E27
T51
45
50
55
60
65
6
5
4
3
^{1}H Chemical Shift [ppm]
^{13}C Chemical Shift [ppm]

Figure S6. Magnified $^{1}H_{\alpha}/^{13}C_{\alpha}$ region in the ^{1}H-detected 2D $^{1}H/^{13}C$ correlation SSNMR spectra of (a) fully protonated microcrystalline GB1, (b) side-chain-selectively deuterated microcrystalline GB1 produced by the inverse fractional deuteration method, and (c) side-chain-deuterated microcrystalline GB1 produced by the solvent-switching method without the deuterated amino-acid mixture. (d–i) Comparison of 1D ^{1}H slices selected at the positions of Glu 27 (^{13}C chemical shift of 59.967 ppm; d, f, h) and Thr 51 (^{13}C chemical shift of 62.571 ppm; e, g, i) for (d, e) fully protonated, (f, g) selectively deuterated by the inverse fractional deuteration method, and (h, i) selectively deuterated by the solvent-witching method without the deuterated amino-acid mixture The spectra were obtained using a 900 MHz JEOL spectrometer with 70 kHz MAS. The 1D and 2D data were processed by NMRPipe/NMRDraw software.

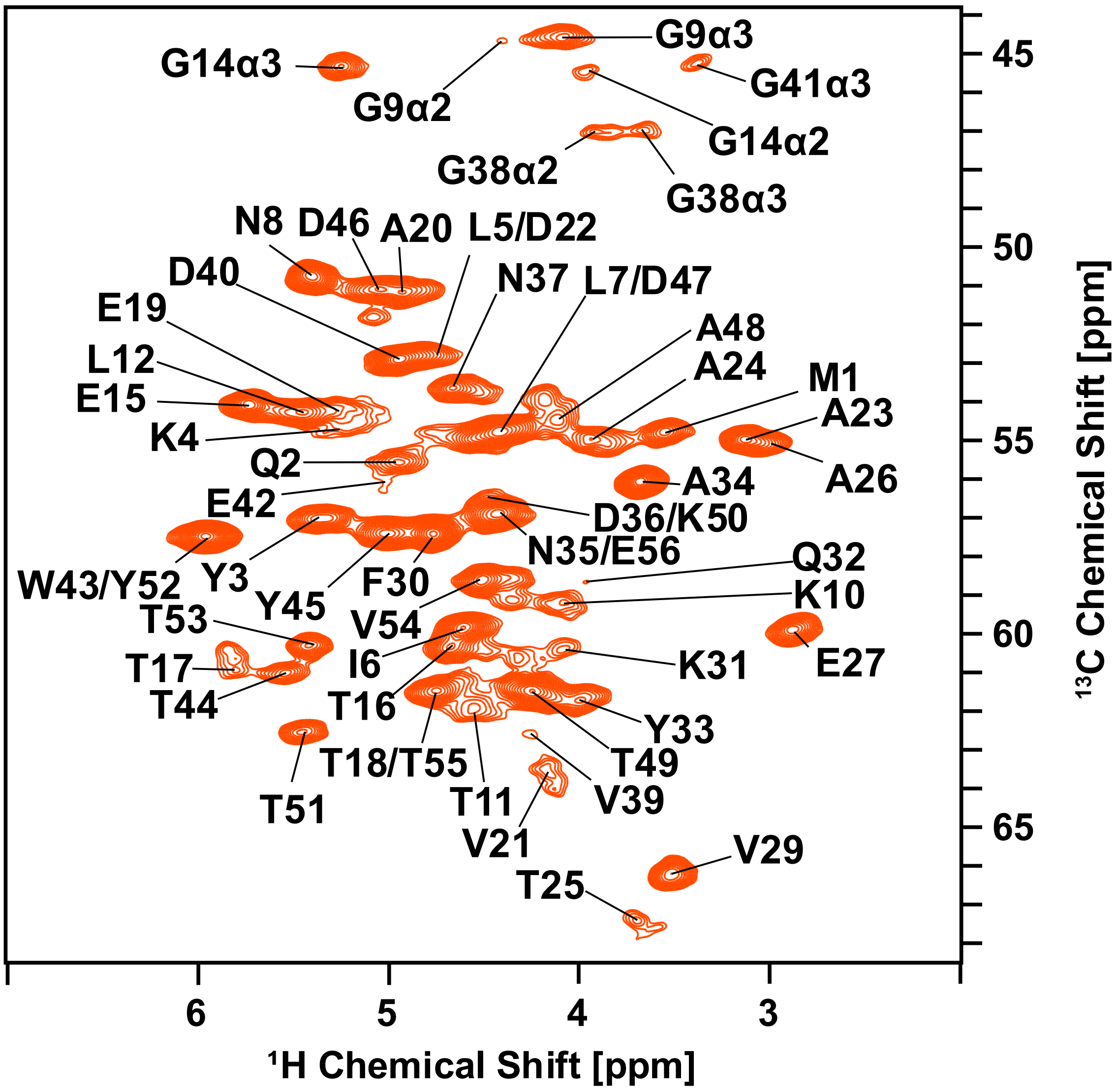

Figure S7. Magnified $^1H_\alpha/^{13}C_\alpha$ region of the ^{1}H-detected 2D $^1H/^{13}C$ correlation SSNMR spectrum of side-chain-selectively deuterated microcrystalline GB1 produced by the solvent-switching method with the deuterated amino-acid mixture. The data, which are the same as those in Fig. 8c, are displayed with full signal assignments.

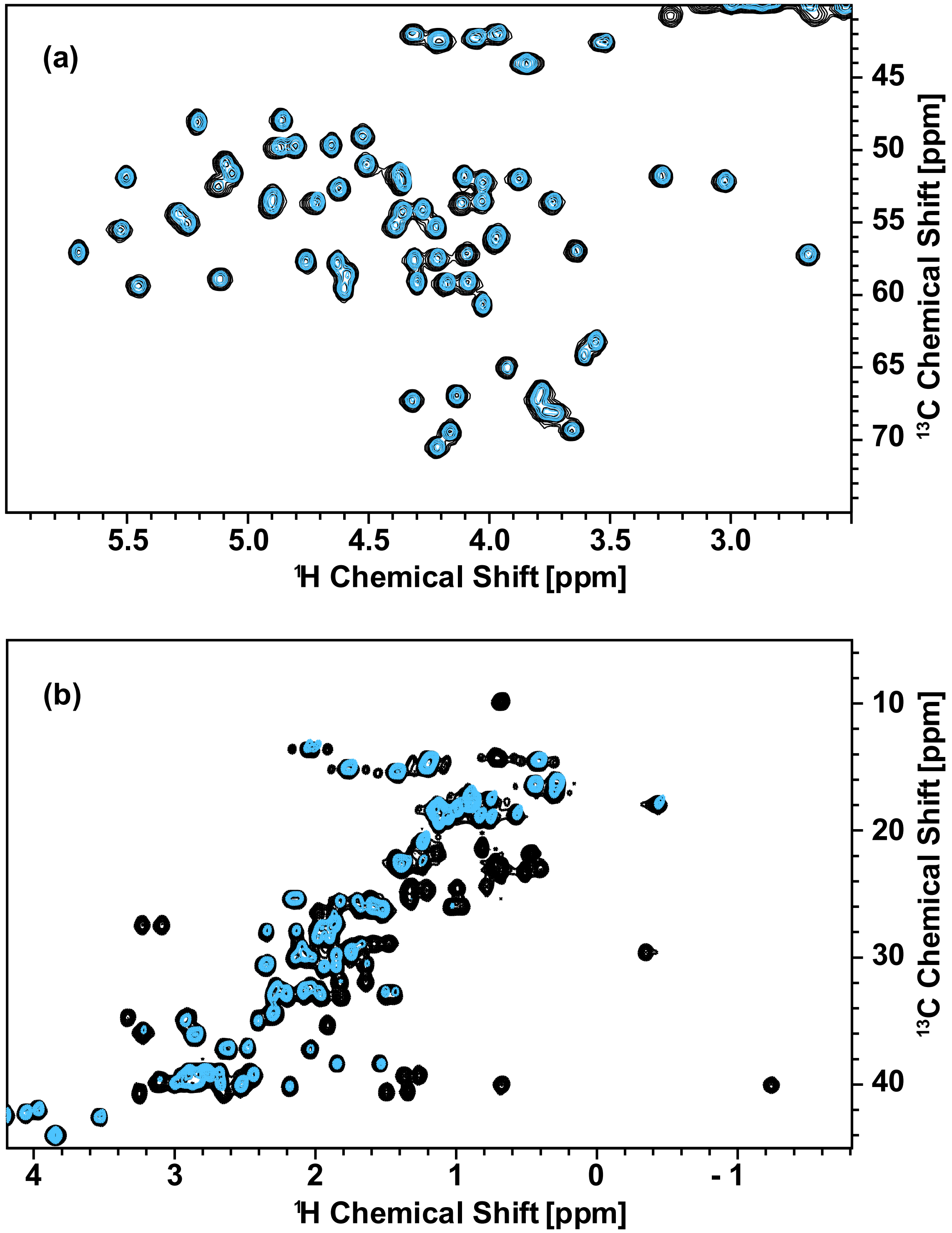

Figure S8. Overlay of ^{1}H-^{13}C HSQC solution NMR spectra of fully protonated GB1 (black) and selectively deuterated GB1 produced with 0.5 g/L of the deuterated amino-acid mixture (light blue) in the (a) $^{1}H_{\alpha}/^{13}C_{\alpha}$ and (b) aliphatic region. The horizontal and vertical axes show the ^{1}H and ^{13}C chemical shifts, respectively. Both samples were dissolved in 50 mM phosphate buffer (pH 5.5), and these spectra were obtained with a

600 MHz solution NMR spectrometer equipped with a cryoprobe. The protein concentrations of fully protonated and selectively deuterated GB1 were 83 and 95 μM, respectively. For a comparison of the signal intensities, counter levels were adjusted based on the protein concentration for each sample.

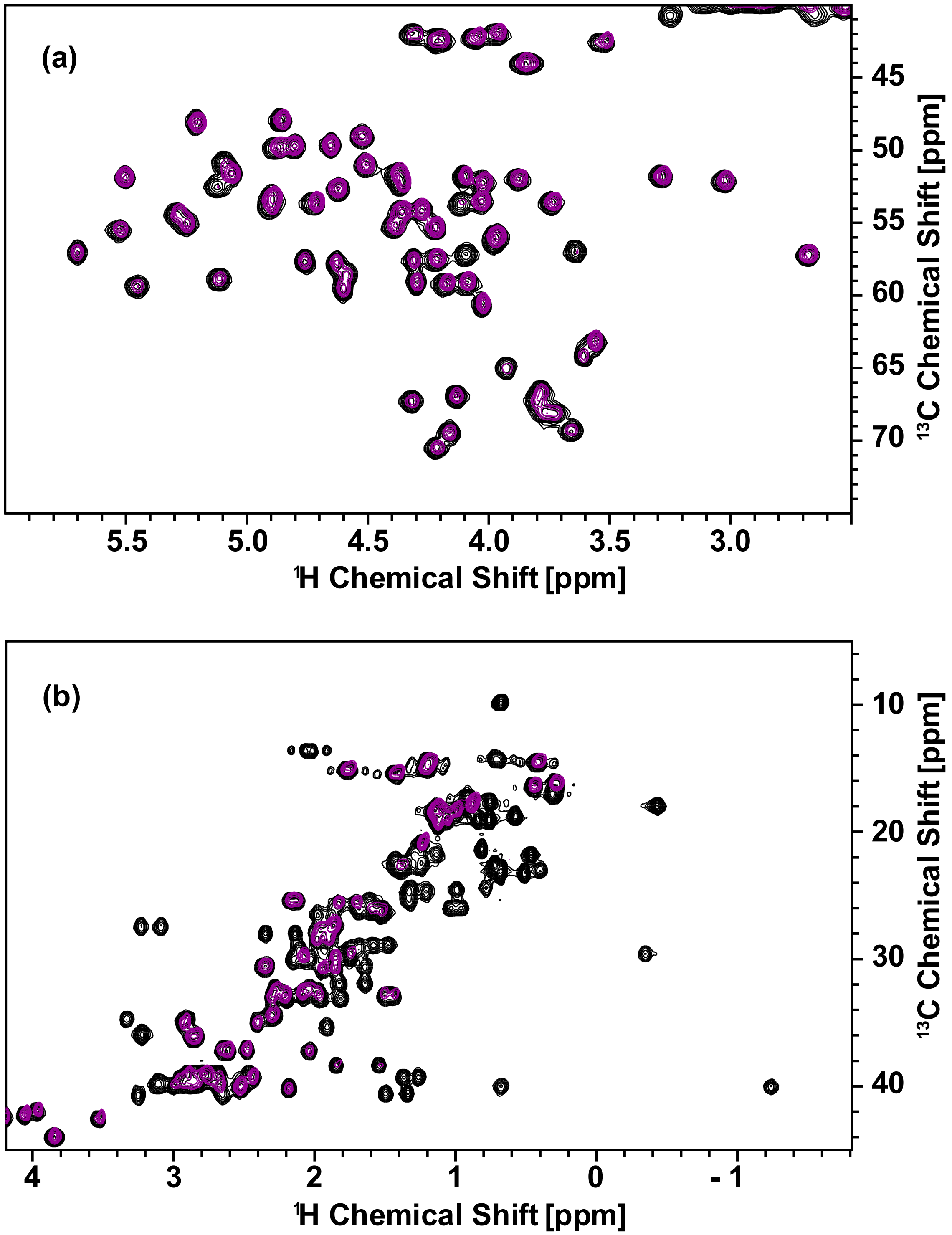

Figure S9. Overlay of ^{1}H-^{13}C HSQC solution NMR spectra of fully protonated GB1 (black) and selectively deuterated GB1 produced with 2.0 g/L of the deuterated amino-acid mixture (purple) in the (a) $^{1}H_{\alpha}/^{13}C_{\alpha}$ and (b) aliphatic region. Both samples were dissolved in 50 mM phosphate buffer (pH 5.5), and these spectra were obtained with a 600 MHz solution NMR spectrometer equipped with a cryoprobe. The fully protonated

and selectively deuterated GB1 protein concentrations were 83 and 86 μM, respectively. For a comparison of the signal intensities, counter levels were adjusted based on the protein concentration for each sample.

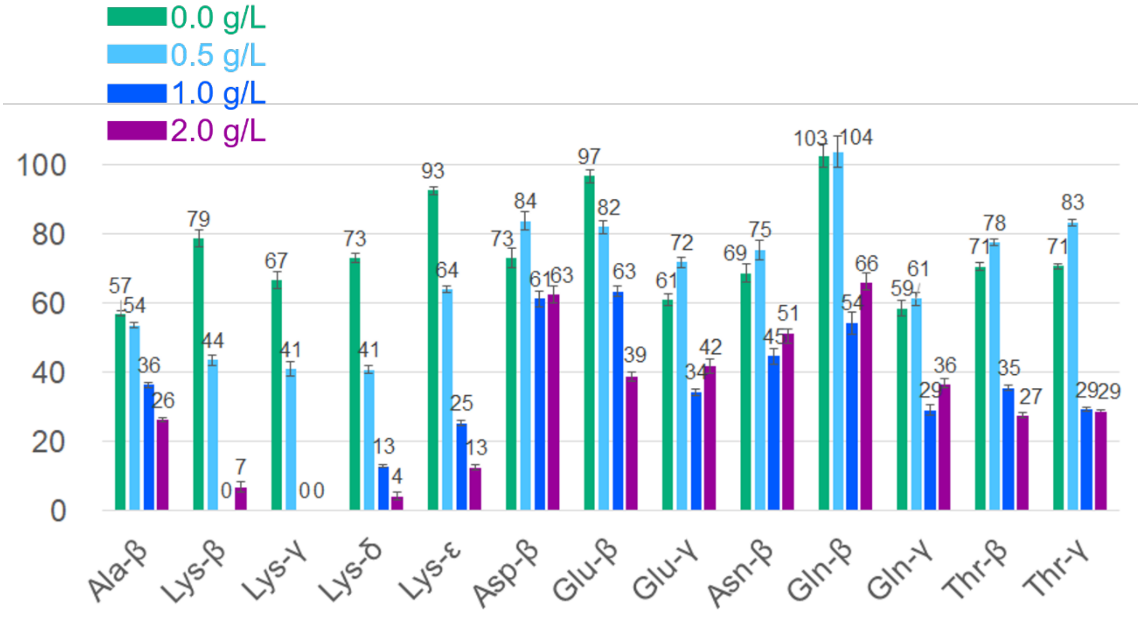

Figure S10. Relative-intensity comparison of side-chain peaks. Green, light blue, blue, and purple filled bars represent solvent-switching deuterated GB1 expressed without or with the amino-acid mixture at concentrations of 0.5, 1.0, and 2.0 g/L, respectively. To adjust for differences in the sample concentrations, the peak intensities of the selectively deuterated samples used in the calculations are normalized by a scaling factor, which is the concentration of the selectively deuterated samples divided by the concentration of the fully protonated sample. Errors were calculated by error propagation with noise.
